# Hierarchical Clustering of Cutaneous Melanoma Based on Immunogenomic Profiling

**DOI:** 10.3389/fonc.2020.580029

**Published:** 2020-11-30

**Authors:** Jie Yu, Minyue Xie, Shengfang Ge, Peiwei Chai, Yixiong Zhou, Jing Ruan

**Affiliations:** Department of Ophthalmology, Shanghai Key Laboratory of Orbital Diseases and Ocular Oncology, Ninth People’s Hospital, Shanghai JiaoTong University School of Medicine, Shanghai, China

**Keywords:** genomic profiling, ssGSEA, immune subtypes, prognosis, cutaneous melanoma

## Abstract

Cutaneous melanoma is an aggressive malignancy with high heterogeneity. Several studies have been performed to identify cutaneous melanoma subtypes based on genomic profiling. However, few classifications based on assessments of immune-associated genes have limited clinical implications for cutaneous melanoma. Using 470 cutaneous melanoma samples from The Cancer Genome Atlas (TCGA), we calculated the enrichment levels of 29 immune-associated gene sets in each sample and hierarchically clustered them into Immunity High (Immunity_H, n=323, 68.7%), Immunity Medium (Immunity_M, n=135, 28.7%), and Immunity Low (Immunity_L, n=12, 2.6%) based on the ssGSEA score. The ESTIMATE algorithm was used to calculate stromal scores (range: -1,800.51–1,901.99), immune scores (range: -1,476.28–3,780.33), estimate scores (range: -2,618.28–5,098.14) and tumor purity (range: 0.216–0.976) and they were significantly correlated with immune subtypes (Kruskal–Wallis test, *P* < 0.001). The Immunity_H group tended to have higher expression levels of HLA and immune checkpoint genes (Kruskal–Wallis test, *P* < 0.05). The Immunity_H group had the highest level of naïve B cells, resting dendritic cells, M1 macrophages, resting NK cells, plasma cells, CD4 memory activated T cells, CD8 T cells, follicular helper T cells and regulatory T cells, and the Immunity_L group had better overall survival. The GO terms identified in the Immunity_H group were mainly immune related. In conclusion, immune signature-associated cutaneous melanoma subtypes play a role in cutaneous melanoma prognosis stratification. The construction of immune signature-associated cutaneous melanoma subtypes predicted possible patient outcomes and provided possible immunotherapy candidates.

## Introduction

Cutaneous melanoma is one of the most aggressive types of cancer due to an elevated degree of heterogeneity in the aspects of clinical presentation, histopathological presentation and genomic profiles (1). Once spread, it becomes life threatening and causes 55,500 deaths every year (2). Due to its heterogeneity, many cutaneous melanoma classification studies have been carried out to lay the foundation for targeted therapies. Akbani R et al. divided 331 cutaneous melanoma patients into four subtypes based on three prevalent significantly mutated genes (BRAF, RAS, and NF1). Though there was no significant clinical correlation with this classification, a subclass whose genome was enriched in immune genes was associated with improved prognosis (3). Zhao Y et al., identified a 25-gene signature that was applied to calculate sample-specific leukocyte infiltration scores (LISs). A higher LIS proved to indicate a better prognosis in metastatic melanoma (4). Nie RC et al. developed an immunoscore based on eight immune subsets (naïve B cells, memory B cells, eosinophils, follicular helper T cells, regulatory T cells, M0 macrophages, plasma cells, and γδT cells), and cutaneous melanoma patients were divided into a high immunoscore group and a low immunoscore group to predict the anti-PD1 response (5). These efforts indicate the importance of classifying cutaneous melanoma for diagnosis and treatment.

To date, there are few treatment options available for cutaneous melanoma. Immunotherapy, such as immune checkpoint blockade, is one of the treatments that has recently increased hope for the survival outcomes of cutaneous melanoma patients (2). However, despite this tremendous advancement, immunotherapeutic strategies exhibit beneficial effects only in a subset of patients. Certain factors, such as tumor genomics, host germline genetics, and the PD-L1 level, influence the responsiveness of immunotherapy (6–8). Tumor microenvironment heterogeneity has been studied as a biomarker for prognosis and immunotherapy sensitivity in various cancers (9, 10). Of note, both infiltrating immune cells and tumor-related stromal cells, which play important roles in tumor growth, progression and drug resistance, are important components of the tumor immune microenvironment (11, 12). Therefore, an increasing number of studies have focused on these factors to provide novel insights into tumor biology and their prognostic value.

In our study, on the basis of immunogenomic profiling, we divided cutaneous melanoma patients into three groups: Immunity High (Immunity_H), Immunity Medium (Immunity_M), and Immunity Low (Immunity_L). We demonstrated that the classification was associated with immune infiltration and survival prognosis. Moreover, we identified subtype-specific genes and Gene Ontology (GO). The construction of immune signature-associated cutaneous melanoma subtypes may help identify possible candidates for immunotherapy.

## Methods

### Database

The transcriptome profiles and clinical data of patients with cutaneous melanoma in this study were downloaded from The Cancer Genome Atlas (TCGA) database (https://portal.gdc.cancer.gov/). In total, 470 cutaneous melanoma patients were enrolled in the current study, and the clinical characteristics included sex, status and TNM stage.

### Single Sample Gene Set Enrichment Analysis (ssGSEA)

For each cutaneous melanoma sample, we quantified the enrichment levels of the 29 immune-associated gene sets, representing immune cell types, functions, and pathways, as described in a previous study (13) by the ssGSEA score. On the basis of the ssGSEA scores of the 29 gene sets, we performed hierarchical clustering of cutaneous melanoma.

### Estimation of STromal and Immune Cells in MAlignant Tumor Tissues Using Expression Data (ESTIMATE)

Stromal scores, immune scores, estimate scores, and the tumor purity of cutaneous melanoma patients were calculated with the ESTIMATE (14) algorithm using the estimate package in R version 3.6.2 (https://www.R-project.org/). All patients were divided into Immunity_H, Immunity_M, and Immunity_L groups.

### Comparison of Immune Cell Infiltration Between Immune Subtypes

The fractions of 22 human immune cell subsets in cutaneous melanoma samples were calculated with Cell-type Identification By Estimating Relative Subsets Of RNA Transcripts (CIBERSORT) (15). One thousand permutations and *P* < 0.05 were set as the criteria to deconvolute each sample. Then, we compared the fractions of the immune cell subsets between immune subtypes with the Mann–Whitney U test.

### Comparison of Survival Prognosis Between Immune Subtypes

With the survival data available, the survival R package was used to analyze the relationship between immune subtypes and the overall survival of patients. The survival differences were compared through a log-rank test, where *P* < 0.05 was regarded as statistically significant. Kaplan–Meier curves were plotted to visualize the differences in survival between immune subtypes.

### Identification of Immune Subtype-Specific GO Terms

To identify the subtype-specific molecular features, we performed a weighted gene co-expression network (16) and identified the gene modules (GO terms) associated with the highly expressed genes in different immune subtypes.

## Results

### Patient Characteristics and Immune Subtype Model Construction

**Figure 1:**
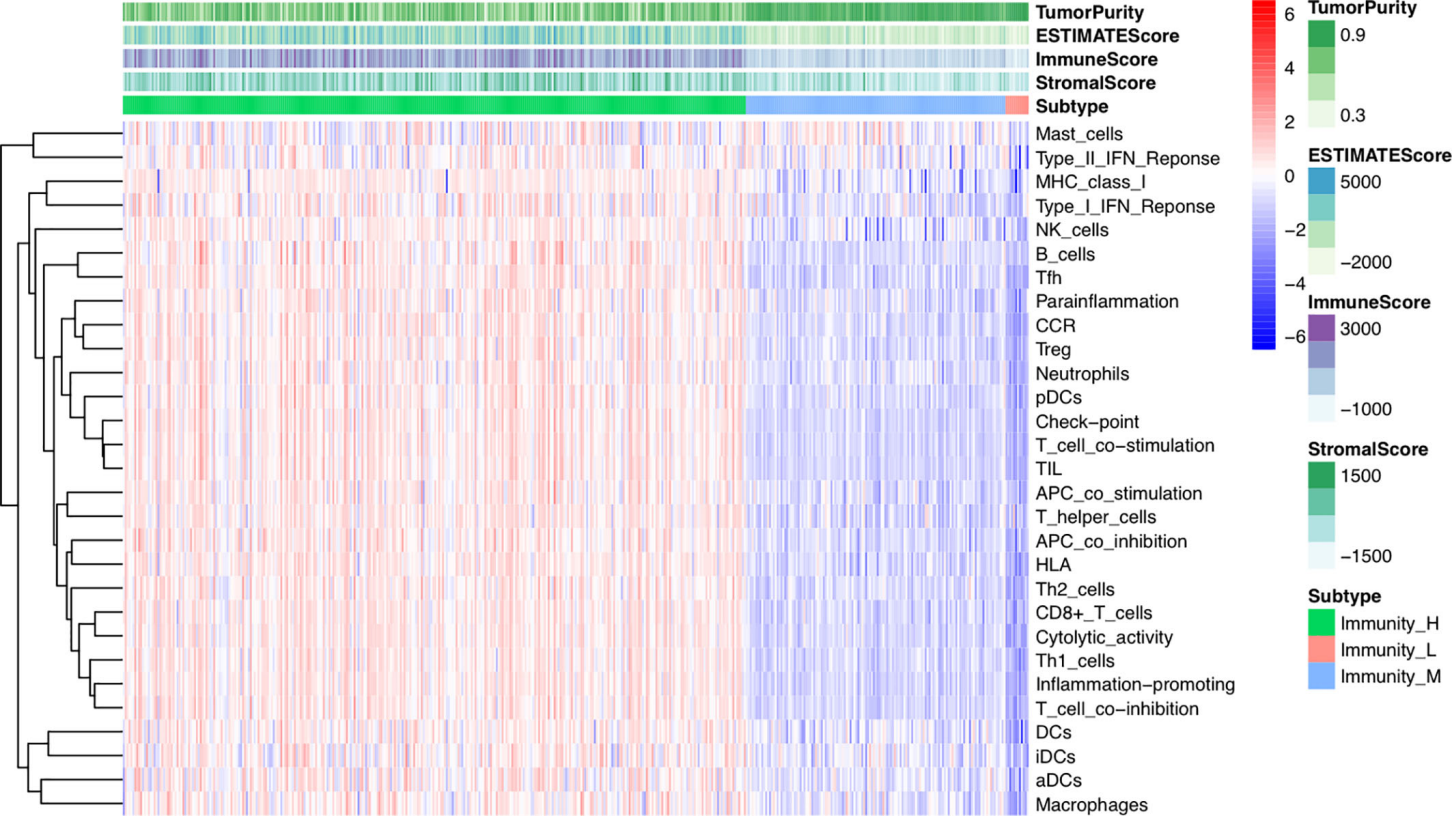
Hierarchical clustering of Cutaneous melanoma into three subtypes. Hierarchical clustering of 470 tumors based on 29 immune-associated gene sets. Immunity_H, Immunity High; Immunity_M, Immunity Medium; Immunity_L, Immunity Low. Tumor purity, estimate scores, stromal scores, and immune scores were evaluated by ESTIMATE.

**Figure 2:**
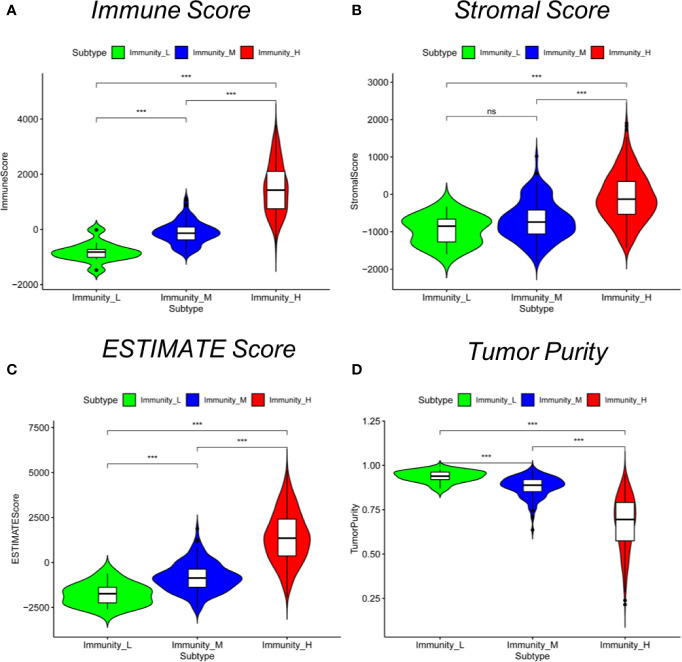
Comparison of stromal scores, immune scores, estimated scores and tumor purity between cutaneous melanoma subtypes. Comparison of **(A)** stromal scores, **(B)** immune scores, **(C)** estimate scores, **(D)** and tumor purity between three subtypes (Mann–Whitney U test). *P < 0.05, ***P < 0.001.

We examined the gene expression profiles and clinical data of 470 cutaneous melanoma patients from TCGA database in this study. Selected patient characteristics are summarized in **Table 1**. The median age at diagnosis was 58.2 (range: 15.0–90.0) years, 290 (61.7%) patients were male, and 211 (44.9%) patients died. We first performed an unsupervised clustering analysis of 29 immune-associated gene sets. Based on the ssGSEA scores of the gene sets, there were three clear groups of samples: Immunity_H (n=323, 68.7%), Immunity_M (n=135, 28.7%) and Immunity_L (n=12, 2.6%) (**Figure 1**). As shown in the heatmap, the Immunity_H group expressed higher levels of immune-associated genes than the Immunity_L group. Cutaneous melanoma patients’ stromal scores (ranging from -1,800.51 to 1,901.99), immune scores (ranging from -1,476.28 to 3,780.33), estimate scores (ranging from -2,618.28 to 5,098.14), and tumor purity (ranging from 0.216 to 0.976) data are shown in **Table S1** (according to the ESTIMATE algorithm). Particular, stromal and immune scores were calculated to predict infiltrating stromal and immune cells levels and to form the basis for the ESTIMATE score to infer tumor purity in tumor tissue (14). We found that the stromal scores, immune scores and estimate scores were significantly high in the Immunity_H group and significantly low in the Immunity_L group (Kruskal–Wallis test, *P* < 0.001) (**Figures 2A–C**), which suggested that these scores were meaningfully correlated with cutaneous melanoma. However, tumor purity showed the opposite trend (Kruskal–Wallis test, P < 0.001) (**Figure 2D**). Notably, these results indicate that Immunity_H samples contain the highest number of immune cells and stromal cells, Immunity_L samples contain the highest number of tumor cells, and Immunity_M samples are somewhere in between.

**Table 1:** Clinical characteristics of the patients.

Characteristic	No. of patients (n = 470) (%)
Age	
median, range	58.2 (15-90)
Gender	
Male	290 (61.7)
Female	180 (38.3)
TNM stage	
I/II NOS	14 (3.0)
0	7 (1.5)
I	77 (16.4)
II	140 (29.8)
III	171 (36.4)
IV	23 (4.9)
Unknown	38 (8.1)
Prior treatment	
None	445 (94.7%)
Neoadjuvant treatment	25 (5.3%)
Survival status	
Death	211 (44.9)
Alive	259 (55.1)

### Immune Subtypes Are Significantly Associated With HLA Genes and Immune Checkpoint Genes

**Figure 3:**
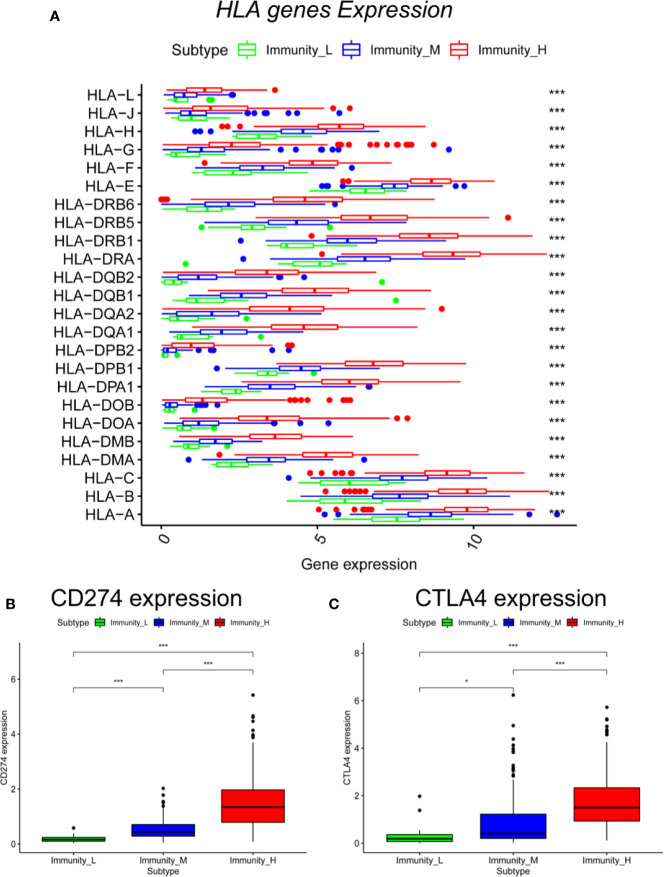
Comparison of HLA genes and immune checkpoint genes between cutaneous melanoma subtypes. Comparison of **(A)** HLA genes (ANOVA test), **(B)** CD274, and **(C)** CTLA4 between three subtypes (Mann–Whitney U test). *P < 0.05, ***P < 0.001.

To test the expression of immune-related genes in each group, we next explored the expression of HLA genes and the immune checkpoint genes in the three immune subtypes. Notably, the expression of all HLA genes was highest in the Immunity_H group and lowest in the Immunity_L group (ANOVA test, *P* < 0.001) (**Figure 3A**). Moreover, the expression levels of programmed cell death 1 ligand (PD-L1), also known as CD274, increased from the Immunity_L group to the Immunity_H group (Immunity_L < Immunity_M < Immunity_H) (Mann–Whitney U test, *P* < 0.001) (**Figure 3B**). The same was true for CTLA4 in the three subtypes (Mann–Whitney U test, *P* < 0.05) (**Figure 3C**). These results showed that these subgroups were significantly associated with the expression of immune-related genes.

### Immune Subtypes Are Significantly Related to Immune Cell Infiltration and Clinical Outcomes

**Figure 4:**
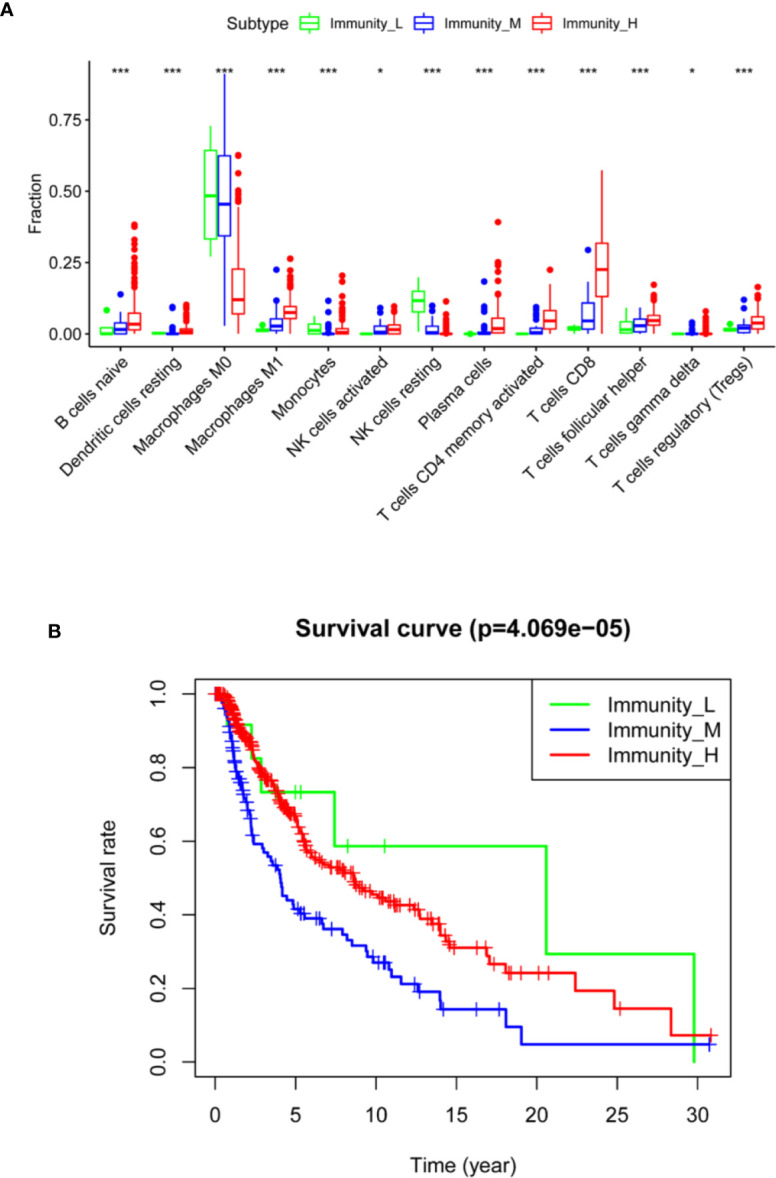
Comparison of immune cell infiltration and clinical outcomes between cutaneous melanoma subtypes. **(A)** Comparison of immune cell infiltration in three subtypes (ANOVA test). **(B)** Comparison of survival prognosis between three subtypes (log-rank test). *P < 0.05, ***P < 0.001.

To further examine the tumor microenvironment, CIBERSORT was applied to assess the proportions of 22 human immune cell subsets in cutaneous melanoma. We found that the Immunity_H group had the highest level of naïve B cells, resting dendritic cells, M1 macrophages, resting NK cells, plasma cells, CD4 memory activated T cells, CD8 T cells, follicular helper T cells, and regulatory T cells, whereas the Immunity_L and Immunity_M groups had relatively low levels of these cell types. In addition, the Immunity_L group had higher levels of M0 macrophages and resting NK cells than the other two subtypes (Mann–Whitney U test, *P* < 0.05) (**Figure 4A**). This result indicated that the Immunity_H group had elevated anti-tumor immune activity.

Next, we investigated the prognostic value of the immune subtypes on patient survival. Interestingly, we found that the Immunity_H and Immunity_M groups had significantly worse overall survival than the Immunity_L group, indicating that these immunological features have distinct clinical outcomes in cutaneous melanoma (**Figure 4B**).

### Identification of Specific GO Terms Associated With the Immune Subtypes

**Figure 5:**
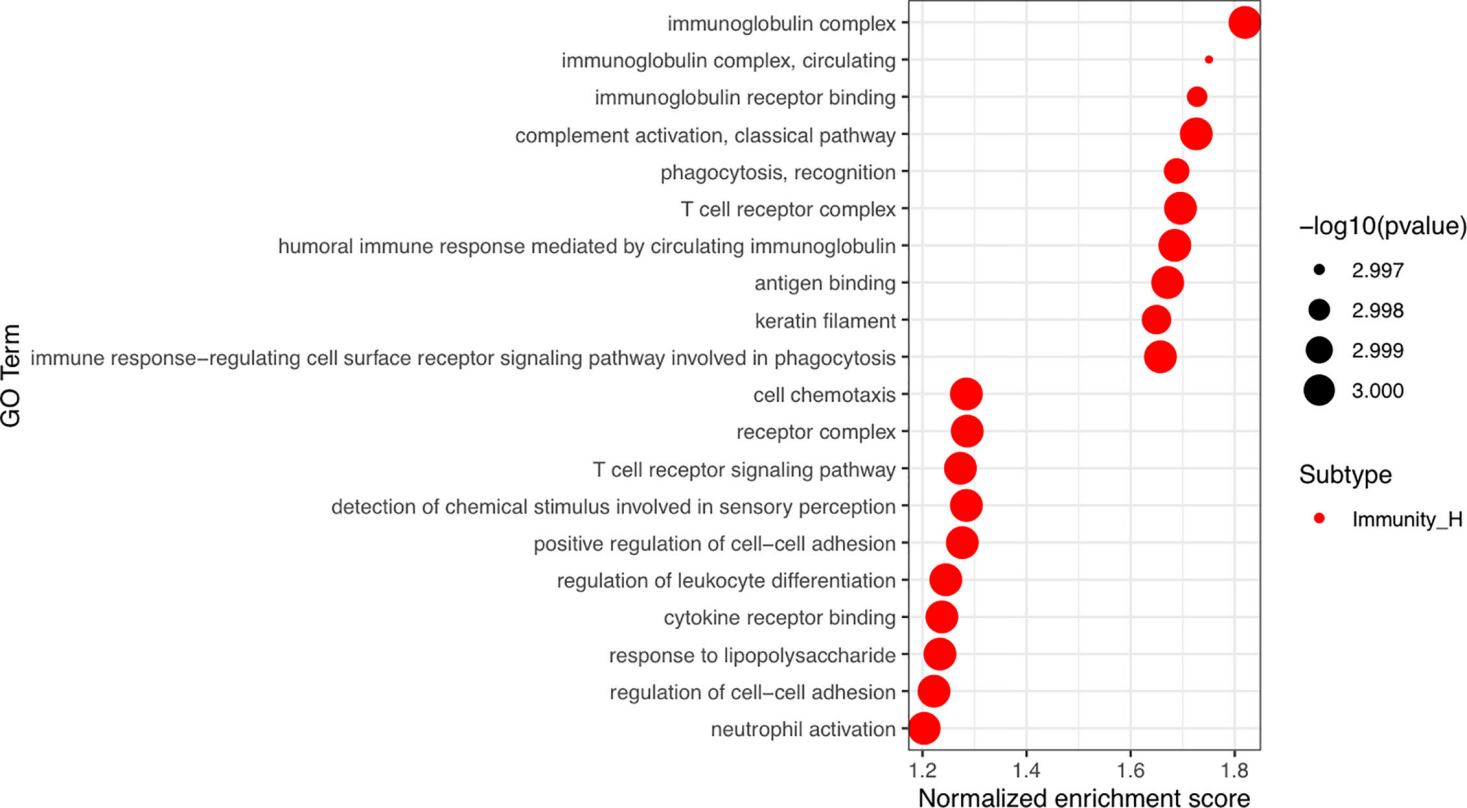
Identification of cutaneous melanoma subtype-specific GO terms. Top 10 GO terms enriched in the Immunity_H and Immunity_L groups. GO, Gene Ontology.

Finally, GSEA was performed to identify a number of GO terms enriched in the Immunity_H and Immunity_L groups. The top 10 GO terms identified in the Immunity_H group were mainly immune related (**Figure 5**, **Table S2**), including immunoglobulin complex; immunoglobulin complex, circulating; immunoglobulin receptor binding; complement activation; classical pathway; T cell receptor complex; humoral immune response mediated by circulating immunoglobulin; antigen binding; and immune response-regulating cell surface receptor signaling pathway involved in phagocytosis. This result also supported elevated immune activity in the Immunity_H group.

## Discussion

Genomic profiling has been used to determine the molecular subtypes in various cancers (17–19), including cutaneous melanoma (3, 4). Currently, accumulating evidence has suggested that the tumor microenvironment plays important roles in tumor progression and therapeutic responses (20, 21). The infiltration of immune cells as well as stromal cells in the tumor microenvironment has an impact on tumor progression and prognosis (22, 23). The development of cutaneous melanoma therapies, especially immunotherapy, has improved clinical outcomes (2). Therefore, an immune-related classification of cutaneous melanoma is needed. Our study found that cutaneous melanoma could be classified into three groups, Immunity_H, Immunity_M, and Immunity_L, using an unsupervised clustering analysis of 29 immune-associated gene sets. Using the ESTIMATE algorithm, we calculated stromal scores, immune scores, estimate scores, and the tumor purity of each patient. We found that stromal scores, immune scores and estimate scores were higher in the Immunity_H group than in the other groups. The Immunity_H group contained more immune cells and stromal cells than the other groups, which suggested elevated immune activity in this subtype. Moreover, GO analysis revealed that a set of gene modules in the Immunity_H group were mainly immune related, including immunoglobulin complex; immunoglobulin complex, circulating; immunoglobulin receptor binding; complement activation; classical pathway; T cell receptor complex; humoral immune response mediated by circulating immunoglobulin; antigen binding; and immune response-regulating cell surface receptor signaling pathway involved in phagocytosis. In the Immunity_L group, cell chemotaxis, receptor complex, detection of chemical stimulus involved in sensory perception, positive regulation of cell−cell adhesion, regulation of leukocyte differentiation, cytokine receptor binding, response to lipopolysaccharide and neutrophil activation. were observed. This further confirmed that immunity was activated in the Immunity_H group.

When we used CIBERSORT to assess the proportions of 22 human immune cell subsets, we found that most immune cells, including naïve B cells, resting dendritic cells, M1 macrophages, resting NK cells, plasma cells, CD4 memory activated T cells, CD8 T cells, follicular helper T cells and regulatory T cells, were significantly higher in the Immunity_H group than in the other groups. We also found that the expression levels of HLA genes and immune checkpoint genes were higher in the Immunity_H group than in the other groups. In addition, the immune checkpoint gene expression levels were significantly associated with the immune subtypes, suggesting that Immunity_H patients may have a good response to anti-PD-L1 or anti-CTLA4 immunotherapy, with evidence that PD-L1 and CTLA4 could serve as biomarkers for corresponding immunotherapeutic responsiveness (24).

The three distinct immune subtypes were strongly associated with clinical outcomes. Numerous studies have demonstrated that enhanced local immune activation contributes to a good prognosis in different kind of tumors (25, 26). In cutaneous melanoma, though several studies have reported that patients with high immune cell infiltration showed better prognosis (27–29), some types of immune cells are associated with worse prognosis, such as CD20-positive tumor‐infiltrating lymphocytes, neutrophil granulocytes and mast cells (30, 31). In our study, based on the immunogenomic profiling of 29 immune signatures, we found that Immunity_L group was associated with better prognosis, which might be the infiltrated immune cells are non-tumor-specific and do not show the anti-tumor effect. Therefore, the underlying mechanism between strong immunogenicity and poor prognosis in cutaneous melanoma needs to be explored.

However, limitations in this study exist. First, it was a retrospective study, and all the data were retrieved from a publicly available database. Thus, external validations are needed to verify our findings. Second, though we identified the immune subtype-specific GO in different groups, further mechanistic studies are encouraged.

## Conclusions

Immune signature-associated cutaneous melanoma subtypes may play a role in cutaneous melanoma prognosis stratification. The construction of immune signature-associated cutaneous melanoma subtypes predicted possible patient outcomes and provided possible candidates for immunotherapy.

## Data Availability Statement

We used publicly available cutaneous melanoma genomic dataset from the TCGA data portal (https://portal.gdc.cancer.gov/). We obtained 29 immune signatures (represented by 29 different gene sets, respectively) from the publications (32, 33).

## Author Contributions

YZ and JY conceptualized and designed the study. JR and YZ were in charge of the financial support. JR was in charge of the administrative support. PC and MX took part in the provision of the study materials and patients. JR and SG collected and assembled the data. JY and PC analyzed and interpreted the data. All authors helped in writing the manuscript. All authors contributed to the article and approved the submitted version.

## Funding

This work was supported by the National Natural Science Foundation of China (No. 81972530, U1932135, 81802702), Fund for Excellent Young Scholars of Shanghai Ninth People’s Hospital, Shanghai Jiao Tong University School of Medicine (JYYQ001), Shanghai Rising-Star Program (17QA1402000), a scholarship from the China Scholarship Council (201906235030), the Science and Technology Commission of Shanghai (17DZ2260100, 19JC1410200) and the pathogenesis and clinical study of orbital disease and eye tumor (SSMU-ZDCX20180400).

## Conflict of Interest

The authors declare that the research was conducted in the absence of any commercial or financial relationships that could be construed as a potential conflict of interest.

## Supplementary Material

Supplementary Figure 1 The mutational landscape of subgroups. Mutational landscape of Immunity_H **(A)** group, Immunity_M **(B)** group, and Immunity_L **(C)** group.

Supplementary Table 1 Stromal scores, immune scores, estimate scores and tumor purity of each sample.

Supplementary Table 2 GO terms enriched in Immunity_H and Immunity_L. GO, gene oncology.

## Abbreviations

CIBERSORT, Cell-type Identification By Estimating Relative Subsets Of RNA Transcripts; ESTIMATE; Estimation of STromal and Immune cells in MAlignant Tumor tissues using Expression data; GO, Gene Ontology; Immunity_H, Immunity High; Immunity_L, Immunity Low; Immunity_M, Immunity Medium; LIS, leukocyte infiltration score.
