# Supplementary figures and images for: Hierarchical Clustering of Cutaneous Melanoma Based on Immunogenomic Profiling
Source: Front Oncol. 2020 Nov 30;10:580029. doi: 10.3389/fonc.2020.580029 (PMC7735560; doi:10.3389/fonc.2020.580029)

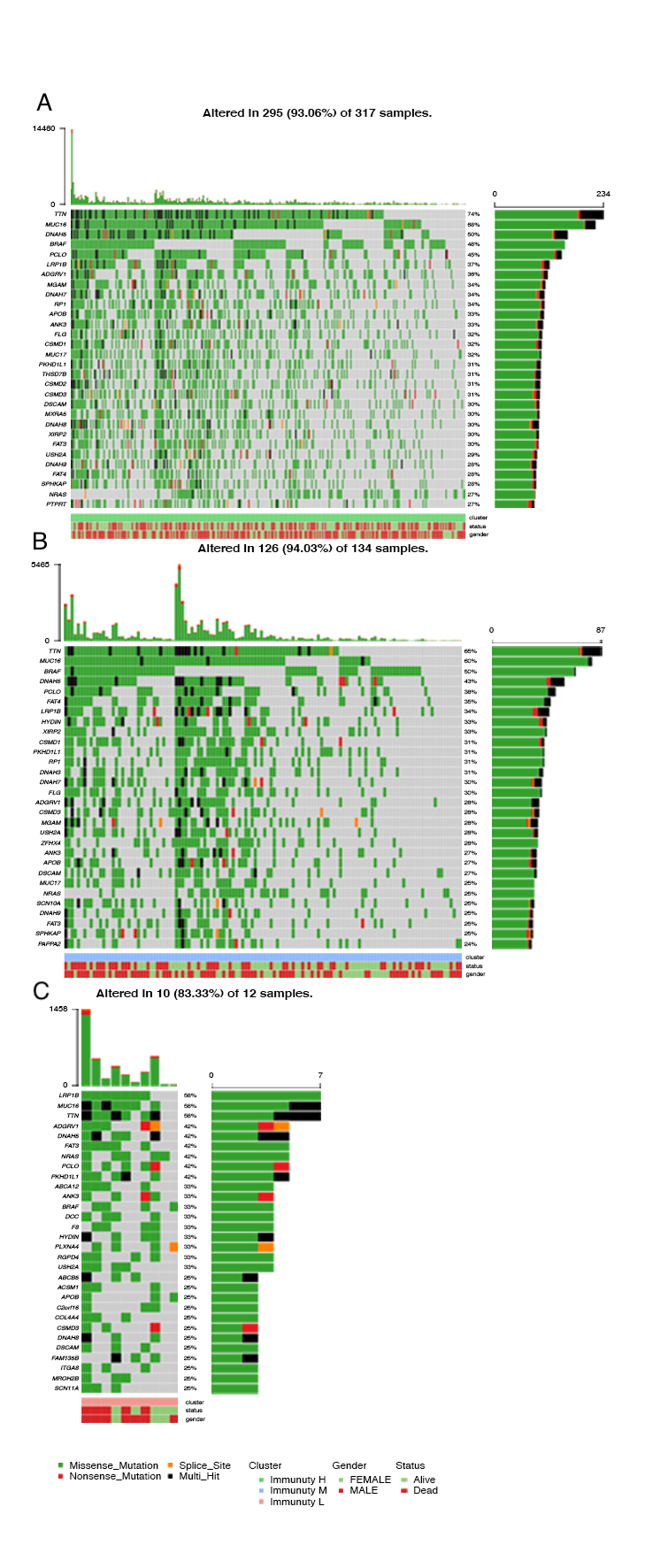

Supplement: Supplementary Figure 1 — The mutational landscape of subgroups. Mutational landscape of Immunity_H (A) group, Immunity_M (B) group, and Immunity_L (C) group. [file Image_1.tif]
