# Supplementary material for: Hierarchical Clustering of Cutaneous Melanoma Based on Immunogenomic Profiling
Source: Front Oncol. 2020 Nov 30;10:580029. doi: 10.3389/fonc.2020.580029 (PMC7735560; doi:10.3389/fonc.2020.580029)
Supplement: Supplementary Table 1 — Stromal scores, immune scores, estimate scores and tumor purity of each sample. [file DataSheet_1.pdf]

Table S1 Stromal scores, immune scores, estimate scores and tumor purity of each sample.

ID	StromalScore	ImmuneScore	ESTIMATEScore	TumorPurity
TCGA-FR-A8YC-06A-11R-A37K-07	-1074.221116	586.0918019	-488.1293145	0.861116077
TCGA-EE-A29P-06A-11R-A18T-07	340.9414115	2509.974313	2850.915725	0.520407221
TCGA-D3-A1Q3-06A-11R-A18T-07	-431.6721013	1448.035144	1016.363043	0.728834942
TCGA-EE-A3JB-06A-11R-A21D-07	519.6556163	1137.90807	1657.563686	0.661260626
TCGA-WE-A8ZY-06A-11R-A37K-07	-484.1344509	296.2733461	-187.8611049	0.837878477
TCGA-EB-A5SE-01A-11R-A311-07	-818.0130193	-222.4984675	-1040.511487	0.899465196
TCGA-HR-A5NC-01A-11R-A27Q-07	-247.07127	67.16973802	-179.901532	0.83724014
TCGA-EE-A2MD-06A-11R-A18T-07	-612.5655192	653.7476241	41.18210492	0.819056235
TCGA-D9-A6EC-06A-11R-A311-07	-1268.689656	-31.98586004	-1300.675516	0.915493658
TCGA-EE-A2MM-06A-11R-A18S-07	-681.1767764	385.9182251	-295.2585513	0.846379274
TCGA-W3-AA1W-06A-11R-A38C-07	-108.880513	3192.601932	3083.721419	0.490927966
TCGA-EE-A29G-06A-12R-A18T-07	-89.57368363	824.3641054	734.7904217	0.756503746
TCGA-D3-A2J8-06A-11R-A18T-07	262.2949491	2702.769342	2965.064291	0.506026855
TCGA-ER-A2NG-06A-11R-A18T-07	-34.78777218	2434.865809	2400.078037	0.575737219
TCGA-D3-A3CC-06A-11R-A18S-07	-26.90740641	296.9617454	270.054339	0.79932333
TCGA-EE-A2MI-06A-11R-A18U-07	277.6239753	1562.285356	1839.909332	0.640947313
TCGA-D3-A3C6-06A-12R-A18U-07	-299.9422608	-272.5367877	-572.4790485	0.867344802
TCGA-D3-A5GS-06A-11R-A27Q-07	158.4413273	1220.904387	1379.345715	0.691336509
TCGA-EB-A6QZ-01A-12R-A32P-07	-924.280954	-136.4921849	-1060.773139	0.900760908
TCGA-EB-A3XE-01A-12R-A239-07	-161.6233862	920.6243102	759.000924	0.754174812
TCGA-EB-A42Y-01A-12R-A24X-07	-412.1716265	255.6160941	-156.5555324	0.835361266
TCGA-D3-A1QA-06A-11R-A18T-07	-831.510421	242.9199501	-588.5904709	0.868519439
TCGA-BF-A5EQ-01A-21R-A27Q-07	306.2012671	1215.50589	1521.707158	0.676087888
TCGA-BF-A1PU-01A-11R-A18S-07	-555.6653773	-799.2536691	-1354.919046	0.918668103
TCGA-WE-A8K4-01A-12R-A37K-07	-826.715908	1707.156487	880.440579	0.74234979
TCGA-ER-A19S-06A-11R-A18U-07	65.75005533	2942.227194	3007.97725	0.500583745
TCGA-D3-A2JE-06A-11R-A37K-07	-1202.06567	-750.9192081	-1952.984878	0.949765155
TCGA-BF-AAP8-01A-11R-A40A-07	-760.3435693	179.0196037	-581.3239655	0.86799026
TCGA-ER-A2NH-06A-11R-A18S-07	691.3000021	2893.840754	3585.140756	0.425534139
TCGA-FR-A726-01A-11R-A32P-07	-774.5403632	-191.1934344	-965.7337976	0.894614462
TCGA-D3-A3C7-06A-11R-A18U-07	-79.79221583	1713.152703	1633.360487	0.663921564
TCGA-EE-A3AH-06A-11R-A18S-07	-454.5996567	-197.6154037	-652.2150605	0.873110592
TCGA-WE-AAA3-06A-11R-A38C-07	537.5902192	1378.805961	1916.39618	0.632289129
TCGA-EB-A97M-01A-11R-A38C-07	-457.1130436	1051.24625	594.1332066	0.769844365
TCGA-ER-A19N-06A-11R-A18S-07	843.7094381	2579.511255	3423.220693	0.446920582
TCGA-D3-A3CB-06A-11R-A18S-07	373.1422758	3011.212366	3384.354642	0.4520169
TCGA-D3-A8GM-06A-11R-A37K-07	-492.4478327	1026.513648	534.0658156	0.775441781
TCGA-BF-A3DM-01A-11R-A20F-07	-908.8083388	-395.729787	-1304.538126	0.915721628
TCGA-DA-A1HW-06A-11R-A18U-07	-214.0476974	1998.128736	1784.081038	0.647216037
TCGA-FW-A3TV-06A-11R-A239-07	-305.693647	671.495707	365.80206	0.790799204
TCGA-ER-A196-01A-11R-A18T-07	-504.7093197	361.0313563	-143.6779634	0.834320686
TCGA-WE-A8K1-06A-21R-A37K-07	-269.957961	1850.493041	1580.53508	0.669700122
TCGA-FS-A1ZZ-06A-11R-A18S-07	-1284.57205	-449.891146	-1734.463196	0.939239555
TCGA-FS-A4F2-06A-11R-A24X-07	-641.3276383	431.9298266	-209.3978117	0.839599925
TCGA-EE-A2GB-06A-11R-A18T-07	-644.8730714	47.60562862	-597.2674428	0.86915004
TCGA-D3-A2JO-06A-11R-A18S-07	-198.0871912	2371.875723	2173.788531	0.602573744

TCGA-Z2-AA3S-06A-11R-A39D-07	-933.3277786	339.7426173	-593.5851613	0.868882601
TCGA-ER-A19C-06A-11R-A18S-07	-177.9973278	200.6927063	22.69537853	0.820610067
TCGA-WE-AA9Y-06A-12R-A38C-07	921.2677049	1112.836279	2034.103984	0.61880948
TCGA-EE-A29X-06A-11R-A18T-07	-175.3865101	1645.894464	1470.507953	0.681606284
TCGA-GF-A3OT-06A-23R-A239-07	391.6698878	1177.659959	1569.329847	0.670920695
TCGA-D9-A1JX-06A-11R-A18S-07	-279.0985934	1580.462308	1301.363715	0.699561764
TCGA-D3-A8GD-06A-11R-A37K-07	-138.1024441	2950.210432	2812.107988	0.525263118
TCGA-DA-A1I7-06A-22R-A18S-07	329.2081019	541.2281559	870.4362578	0.743332914
TCGA-FS-A4FB-06A-11R-A266-07	-607.9725872	-521.7323533	-1129.704941	0.905109294
TCGA-ER-A3ET-06A-11R-A20F-07	639.6878262	1220.345644	1860.03347	0.638677082
TCGA-D9-A3Z1-06A-11R-A239-07	-153.2376528	2108.009322	1954.771669	0.627914992
TCGA-ER-A1A1-06A-11R-A18U-07	316.7163374	3304.326871	3621.043208	0.420759148
TCGA-FS-A4F8-06A-11R-A266-07	783.8166706	2672.549148	3456.365818	0.442562923
TCGA-DA-A1I0-06A-11R-A20F-07	-120.769467	1518.302179	1397.532712	0.689405144
TCGA-EE-A2MJ-06A-11R-A18S-07	101.8648599	2518.472631	2620.33749	0.549005547
TCGA-BF-AAP6-01A-11R-A40A-07	-773.7430703	-34.24930236	-807.9923727	0.8840292
TCGA-ER-A2NF-01A-11R-A18T-07	433.437093	1381.722477	1815.15957	0.643731696
TCGA-D3-A1Q4-06A-11R-A18T-07	1432.273929	455.5413135	1887.815242	0.635533829
TCGA-3N-A9WB-06A-11R-A38C-07	-1312.6348	-580.615914	-1893.250714	0.946984528
TCGA-EE-A2GO-06A-11R-A18S-07	-748.9080505	-239.0707063	-987.9787568	0.896068754
TCGA-EE-A3AG-06A-31R-A18S-07	-1064.594945	-463.3725647	-1527.96751	0.928405025
TCGA-DA-A3F3-06A-11R-A20F-07	-720.0184892	95.87138814	-624.1471011	0.871094571
TCGA-EB-A5SG-06A-11R-A311-07	694.2204334	3104.244707	3798.465141	0.396993206
TCGA-W3-AA21-06A-11R-A38C-07	-1281.35032	119.3432898	-1162.007031	0.907115129
TCGA-BF-A1Q0-01A-21R-A18S-07	-801.0664728	695.8055722	-105.2609007	0.831198673
TCGA-FS-A1ZQ-06A-11R-A18U-07	-739.4754758	463.0634168	-276.4120589	0.844902681
TCGA-EE-A2GP-06A-11R-A18S-07	644.7824858	1045.298244	1690.08073	0.657672518
TCGA-FS-A1ZR-06A-21R-A18U-07	-92.09875873	367.1515742	275.0528154	0.798882222
TCGA-OD-A75X-06A-12R-A32P-07	-922.2771316	192.478597	-729.7985347	0.878605929
TCGA-DA-A95Y-06A-11R-A37K-07	-1168.124504	86.69717857	-1081.427326	0.902073522
TCGA-EE-A29L-06A-12R-A18S-07	-1800.507848	-817.7730668	-2618.280915	0.975754533
TCGA-D3-A3CE-06A-11R-A18S-07	196.8635153	1768.254111	1965.117626	0.626732318
TCGA-EE-A3AF-06A-11R-A18S-07	-63.05019181	1362.968593	1299.918401	0.699713348
TCGA-D3-A3C3-06A-12R-A18S-07	-691.4104299	1395.276643	703.8662127	0.759464605
TCGA-EE-A3J5-06A-11R-A20F-07	118.8068127	1429.388065	1548.194878	0.673217959
TCGA-XV-A9W5-01A-11R-A38C-07	-316.5642737	442.7008622	126.1365885	0.811838341
TCGA-GN-A8LL-06A-21R-A37K-07	-962.6208924	-78.76753111	-1041.388424	0.89952144
TCGA-EB-A3XF-01A-31R-A239-07	-565.6556033	933.8310618	368.1754585	0.790585917
TCGA-EB-A6R0-01A-12R-A32P-07	-837.4411785	1365.115187	527.674008	0.776033864
TCGA-FS-A4F9-06A-11R-A24X-07	-1208.783334	-31.12521081	-1239.908544	0.911868526
TCGA-FS-A1YY-06A-11R-A18T-07	191.2211516	39.46724553	230.6883971	0.802782251
TCGA-EE-A2GH-06A-11R-A18T-07	967.4203123	2594.56556	3561.985873	0.428607455
TCGA-GF-A2C7-01A-11R-A18T-07	-733.0012449	-601.46194	-1334.463185	0.917477813
TCGA-EE-A2GT-06A-12R-A18S-07	-207.6696968	2166.240636	1958.570939	0.627480856
TCGA-EB-A51B-01A-11R-A27Q-07	-1422.712774	296.8896499	-1125.823124	0.904866878
TCGA-EE-A29R-06A-11R-A18T-07	-395.2663476	-320.7676769	-716.0340245	0.877639266
TCGA-ER-A19E-06A-11R-A18S-07	-302.1387637	281.8356988	-20.30306488	0.824200746
TCGA-HR-A2OH-06A-11R-A18U-07	194.3428936	3167.09769	3361.440584	0.455014627

TCGA-GN-A4U7-06A-21R-A32P-07	-1360.912386	-497.3637555	-1858.276141	0.945322653
TCGA-EB-A42Z-01A-12R-A24X-07	-261.0984302	419.371591	158.2731608	0.809074984
TCGA-ER-A42H-01A-11R-A24X-07	-238.8946414	-223.3824387	-462.2770801	0.859180569
TCGA-FS-A1ZA-06A-11R-A18T-07	-530.2327785	759.0934941	228.8607156	0.802942192
TCGA-DA-A1HY-06A-11R-A18T-07	-58.80301375	56.61323507	-2.189778679	0.82269215
TCGA-EB-A3Y6-01A-21R-A239-07	58.28460961	507.8032106	566.0878202	0.772465266
TCGA-FR-A7U9-06A-11R-A352-07	904.1297913	1994.316487	2898.446278	0.514436873
TCGA-EB-A3XC-01A-11R-A239-07	-419.3600862	545.3861293	126.0260431	0.811847815
TCGA-ER-A42L-06A-11R-A24X-07	699.2430414	2654.332615	3353.575656	0.45604237
TCGA-ER-A19M-06A-61R-A239-07	553.9210551	1866.318459	2420.239515	0.573314934
TCGA-FS-A1ZD-06A-11R-A18T-07	-8.523752779	54.8685529	46.34480012	0.818621226
TCGA-ER-A19G-06A-11R-A18U-07	-248.0412299	1902.152421	1654.111191	0.66164071
TCGA-WE-A8ZQ-06A-41R-A37K-07	-628.9701644	-773.959551	-1402.929715	0.921429207
TCGA-QB-AA9O-06A-11R-A39D-07	-654.2875303	1534.858763	880.5712324	0.74233694
TCGA-ER-A19T-06A-11R-A18U-07	-171.685062	1064.840389	893.155327	0.741098001
TCGA-ER-A2NF-06A-11R-A18T-07	-939.7557699	888.0727397	-51.68303018	0.826800482
TCGA-BF-A5ER-01A-12R-A27Q-07	-677.4750604	10.32448996	-667.1505704	0.874177317
TCGA-ER-A2NC-06A-11R-A18T-07	804.1432735	1380.469285	2184.612558	0.601304987
TCGA-D3-A1QA-07A-11R-A37K-07	-936.9631666	631.6386536	-305.324513	0.847165273
TCGA-DA-A95V-06A-11R-A37K-07	-490.1448646	1423.465516	933.3206516	0.737126719
TCGA-EB-A82B-01A-11R-A352-07	-372.2659902	1346.538539	974.2725486	0.733051287
TCGA-EB-A5VV-06A-11R-A32P-07	592.3538393	3780.29744	4372.65128	0.318317975
TCGA-EE-A2A5-06A-11R-A18T-07	-139.2467296	-98.82089867	-238.0676283	0.841878507
TCGA-EE-A29D-06A-11R-A18T-07	-924.5273432	-272.6377328	-1197.165076	0.909275127
TCGA-EE-A3J7-06A-11R-A20F-07	-1212.518924	-185.3377251	-1397.856649	0.921139614
TCGA-EE-A2MQ-06A-11R-A18S-07	-97.6225473	646.7865684	549.1640211	0.774040501
TCGA-BF-AAP7-01A-11R-A40A-07	320.3203931	2100.652896	2420.973289	0.573226681
TCGA-EE-A29A-06A-12R-A18U-07	72.81269864	-659.7310005	-586.9183018	0.868397752
TCGA-DA-A1IB-06A-11R-A18S-07	487.0141309	3444.698296	3931.712426	0.378966602
TCGA-XV-A9W2-01A-11R-A39D-07	-1039.451079	7.643552764	-1031.807526	0.898906143
TCGA-D9-A3Z4-01A-11R-A239-07	-98.93513969	1121.402208	1022.467069	0.728221168
TCGA-EE-A2GJ-06A-11R-A18U-07	-283.058672	1690.769057	1407.710385	0.688322184
TCGA-EB-A4P0-01A-41R-A266-07	-408.1500364	393.6967518	-14.4532846	0.823714173
TCGA-EB-A3HV-01A-11R-A21D-07	167.0277033	683.2400135	850.2677169	0.745310002
TCGA-EE-A2GK-06A-11R-A18S-07	585.3424244	3616.806301	4202.148726	0.341941759
TCGA-WE-A8JZ-06A-11R-A37K-07	-705.6905998	-82.3680522	-788.058652	0.882657694
TCGA-EE-A2MP-06A-11R-A18S-07	-295.5013809	1408.742901	1113.24152	0.719024857
TCGA-D3-A5GO-06A-12R-A27Q-07	-280.0857161	1418.661839	1138.576123	0.716435365
TCGA-D3-A51J-06A-11R-A266-07	191.9753991	2373.726632	2565.702031	0.555690962
TCGA-QB-A6FS-06A-11R-A311-07	58.11171065	1952.486498	2010.598208	0.621516203
TCGA-EB-A4XL-01A-11R-A27Q-07	-258.670192	849.9175748	591.2473828	0.770114654
TCGA-GN-A264-06A-11R-A18U-07	118.6769372	137.9949938	256.671931	0.800502186
TCGA-D3-A2JF-06A-11R-A18S-07	-47.27887501	1820.865077	1773.586202	0.648389617
TCGA-EE-A3JD-06A-11R-A20F-07	327.7826798	2964.510193	3292.292873	0.464029518
TCGA-DA-A1HV-06A-21R-A18S-07	-498.2762085	2064.451431	1566.175223	0.671263997
TCGA-D9-A4Z2-01A-11R-A24X-07	-261.5333924	59.58247232	-201.9509201	0.839005636
TCGA-DA-A1I8-06A-11R-A18T-07	-530.3676285	581.6199604	51.25233186	0.818207282
TCGA-D3-A5GN-06A-11R-A27Q-07	52.859413	857.9896585	910.8490715	0.739351727

TCGA-FS-A1ZS-06A-12R-A18T-07	222.2505299	537.0113944	759.2619243	0.754149654
TCGA-ER-A19J-06A-11R-A18S-07	306.8453783	377.814601	684.6599793	0.761295645
TCGA-EE-A2MK-06A-11R-A18S-07	52.97446412	1679.926231	1732.900695	0.652924685
TCGA-BF-AAOX-01A-11R-A39D-07	-1322.452747	-200.3137176	-1522.766464	0.928121079
TCGA-FR-A728-01A-11R-A32P-07	25.32397723	1890.885459	1916.209436	0.632310365
TCGA-EE-A2GS-06A-12R-A18S-07	-223.6783331	720.6910075	497.0126744	0.778864565
TCGA-YG-AA3O-06A-11R-A38C-07	-499.1249412	410.2376978	-88.88724343	0.829860007
TCGA-EE-A2A2-06A-11R-A18T-07	-933.6729076	991.3927321	57.71982454	0.817661108
TCGA-EE-A2ME-06A-11R-A18T-07	1170.544784	3369.902754	4540.447538	0.294874423
TCGA-EE-A29C-06A-21R-A18S-07	506.1072077	494.3555731	1000.462781	0.730431003
TCGA-EB-A4OY-01A-11R-A266-07	-815.4809818	717.304182	-98.1767998	0.830620085
TCGA-3N-A9WC-06A-11R-A38C-07	-78.08541033	2209.922392	2131.836981	0.607476761
TCGA-ER-A3PL-06A-11R-A239-07	197.1952101	344.77044	541.9656501	0.774709064
TCGA-BF-AAP0-06A-11R-A39D-07	571.744514	2582.159055	3153.903569	0.481927035
TCGA-ER-A19B-06A-11R-A18S-07	-499.892535	108.8122075	-391.0803275	0.853786331
TCGA-WE-A8ZO-06A-11R-A37K-07	-498.6906435	1395.635976	896.9453327	0.740724369
TCGA-FS-A1Z3-06A-11R-A18T-07	-538.9266639	-157.0681978	-695.9948617	0.876225538
TCGA-EE-A20B-06A-11R-A18U-07	466.7415579	1090.702881	1557.444439	0.672213374
TCGA-FS-A4F5-06A-11R-A266-07	-737.5985317	-79.63193532	-817.230467	0.884662245
TCGA-EE-A29W-06A-11R-A18U-07	557.6535203	-384.3475503	173.30597	0.80777616
TCGA-EE-A29B-06A-11R-A18U-07	-851.7314557	489.3389294	-362.3925264	0.851586402
TCGA-D3-A51E-06A-11R-A266-07	-620.8961902	1606.095464	985.1992737	0.731959403
TCGA-FS-A1ZF-06A-12R-A18S-07	50.615323	-212.447042	-161.831719	0.835786749
TCGA-WE-A8ZM-06A-11R-A37K-07	-330.2768588	-789.8122929	-1120.089152	0.904508259
TCGA-LH-A9QB-06A-11R-A38C-07	-510.7298728	-230.6254437	-741.3553165	0.879414777
TCGA-RP-A693-06A-13R-A311-07	-60.4552796	1578.079493	1517.624214	0.676529365
TCGA-D3-A51H-06A-12R-A266-07	105.4603147	3353.656504	3459.116818	0.44220077
TCGA-WE-AAA4-06A-12R-A38C-07	581.625949	2829.028639	3410.654588	0.448569914
TCGA-EE-A181-06A-11R-A18S-07	-586.8585026	1975.390931	1388.532429	0.690361543
TCGA-WE-A8ZR-06A-11R-A37K-07	-1145.066291	349.3689818	-795.6973088	0.883184152
TCGA-EB-A5FP-01A-11R-A27Q-07	-1374.265682	-557.3598926	-1931.625574	0.948779255
TCGA-EE-A182-06A-11R-A18T-07	123.9729728	1119.727693	1243.700666	0.705584961
TCGA-D9-A148-06A-11R-A18S-07	-563.750147	707.8843184	144.1341715	0.810292988
TCGA-FR-A7UA-06A-32R-A352-07	698.8183009	3580.010802	4278.829103	0.331343018
TCGA-D3-A1Q6-06A-11R-A18T-07	-987.5435236	477.4260667	-510.117457	0.86275253
TCGA-WE-A8ZT-06A-11R-A37K-07	-1190.373352	-149.8933971	-1340.266749	0.917816353
TCGA-D3-A2JC-06A-11R-A18T-07	-182.7557393	1512.93761	1330.181871	0.696532751
TCGA-FS-A1YX-06A-11R-A18T-07	102.3083375	459.7397865	562.0481241	0.772841707
TCGA-EB-A6L9-06A-11R-A32P-07	253.906703	2532.04885	2785.955553	0.528525891
TCGA-EE-A29S-06A-11R-A18T-07	110.8371147	903.5797	1014.416815	0.729030517
TCGA-EE-A17Y-06A-11R-A18T-07	-430.1159585	-356.7362754	-786.8522339	0.882574446
TCGA-FS-A1ZJ-06A-12R-A18S-07	-817.0465779	119.6944144	-697.3521635	0.876321533
TCGA-BF-A1PV-01A-11R-A18U-07	-1065.499225	-678.2143188	-1743.713544	0.939704786
TCGA-ER-A19W-06A-41R-A239-07	506.7391976	3055.584038	3562.323236	0.428562712
TCGA-EE-A2GM-06B-11R-A18S-07	-843.4540782	4.129455445	-839.3246228	0.886169659
TCGA-EE-A2MU-06A-21R-A18S-07	504.4181269	2275.322797	2779.740924	0.529300083
TCGA-D3-A8GS-06A-12R-A37K-07	-797.2638801	2125.69926	1328.43538	0.696716676
TCGA-EB-A4IS-01A-21R-A266-07	352.3096409	2176.113043	2528.422684	0.560232126

TCGA-W3-A824-06A-21R-A352-07	-680.4127848	1668.597118	988.1843333	0.731660785
TCGA-W3-AA1V-06B-11R-A40A-07	-91.80253247	1273.655895	1181.853362	0.711989028
TCGA-ER-A19O-06A-11R-A18S-07	948.6973963	2591.785417	3540.482814	0.431457093
TCGA-GN-A265-06A-21R-A18T-07	1418.327459	2573.419194	3991.746653	0.370796973
TCGA-EE-A20H-06A-11R-A18S-07	-980.6901039	-702.8907657	-1683.58087	0.936649577
TCGA-FS-A1Z0-06A-11R-A18T-07	-41.06435089	1702.275229	1661.210878	0.660858924
TCGA-D3-A3MO-06A-11R-A21D-07	-627.3405246	-215.8440274	-843.1845521	0.886432054
TCGA-W3-AA1R-06A-11R-A39D-07	-1436.226111	532.5802319	-903.6458793	0.890504971
TCGA-D9-A1JW-06A-11R-A18S-07	-355.2583543	1890.816289	1535.557935	0.674588437
TCGA-ER-A2NB-01A-12R-A18S-07	-245.2876248	1600.779634	1355.492009	0.693862177
TCGA-EE-A29Q-06A-11R-A18T-07	-1098.215032	-209.6256624	-1307.840695	0.915916311
TCGA-EB-A44R-06A-41R-A266-07	-252.2566019	823.9019122	571.6453103	0.771946946
TCGA-DA-A95W-06A-11R-A37K-07	-990.6001716	581.7722701	-408.8279014	0.855139728
TCGA-EE-A29T-06A-11R-A18T-07	13.2218343	1772.8638	1786.085634	0.646991699
TCGA-DA-A1I5-06A-11R-A18T-07	634.391585	2270.42716	2904.818745	0.513634512
TCGA-FS-A1Z7-06A-11R-A18T-07	1020.307992	862.9620296	1883.270022	0.636048803
TCGA-EE-A20F-06A-21R-A18S-07	-372.8323337	1306.177478	933.3451442	0.73712429
TCGA-RP-A694-06A-11R-A311-07	-486.0316268	778.4546199	292.4229932	0.79734599
TCGA-FR-A729-06A-11R-A352-07	-54.91179155	2358.059308	2303.147516	0.587312084
TCGA-EE-A2M5-06A-12R-A18S-07	-529.4285894	1278.856252	749.4276626	0.755096854
TCGA-BF-AAP4-01A-11R-A40A-07	-325.8053573	1431.485165	1105.679808	0.719795827
TCGA-D3-A3ML-06A-11R-A21D-07	-1512.088443	-916.6853257	-2428.773768	0.969289475
TCGA-ER-A42K-06A-11R-A24X-07	-465.0070661	152.0602103	-312.9468558	0.847759232
TCGA-EB-A3XD-01A-22R-A239-07	543.4400445	1686.145382	2229.585427	0.596017199
TCGA-ER-A2ND-06A-11R-A18T-07	-915.9302796	553.4373568	-362.4929228	0.851594128
TCGA-EE-A2MH-06A-11R-A18S-07	-331.1156948	1973.134831	1642.019136	0.66297058
TCGA-EB-A4IQ-01A-12R-A266-07	-1188.682194	-190.0064918	-1378.688686	0.920040812
TCGA-D3-A3C1-06A-12R-A18S-07	-530.882869	881.3568802	350.4740112	0.792174359
TCGA-ER-A19K-01A-21R-A18T-07	-669.4803612	679.3096774	9.829316184	0.821687909
TCGA-FW-A3I3-06A-11R-A21D-07	-1366.065471	-437.4789468	-1803.544418	0.942671998
TCGA-EE-A2GD-06A-11R-A18T-07	1128.397645	1127.116401	2255.514046	0.592956763
TCGA-EB-A5SF-01A-11R-A311-07	-217.772087	188.7623055	-29.00978148	0.824923826
TCGA-ER-A195-06A-11R-A18U-07	1018.611655	2113.454577	3132.066232	0.484733221
TCGA-EB-A553-01A-12R-A27Q-07	-303.1388466	1044.7088	741.5699533	0.755852549
TCGA-EB-A82C-01A-11R-A352-07	-952.365741	-620.5580136	-1572.923755	0.930836802
TCGA-D3-A3C8-06A-12R-A18S-07	873.5449956	3473.658502	4347.203498	0.321856883
TCGA-EB-A44O-01A-11R-A266-07	-1129.848968	-154.4330022	-1284.28197	0.914522843
TCGA-EE-A185-06A-11R-A18S-07	-1530.764128	-842.870577	-2373.634705	0.967267306
TCGA-D3-A2JN-06A-11R-A18S-07	436.3246039	1588.722702	2025.047306	0.619853243
TCGA-FS-A1ZH-06A-11R-A18T-07	862.2353236	2033.146202	2895.381526	0.514822597
TCGA-XV-A9VZ-01A-11R-A38C-07	-510.4555638	-634.2432144	-1144.698778	0.906042887
TCGA-ER-A19L-06A-12R-A18S-07	229.7658457	29.69412306	259.4599688	0.800256843
TCGA-FS-A1ZG-06A-11R-A18T-07	-1259.301981	-399.7547902	-1659.056771	0.935382598
TCGA-FR-A3YO-06A-11R-A239-07	-183.9592048	894.5706902	710.6114855	0.758820107
TCGA-W3-A828-06A-11R-A352-07	1252.58457	751.5711964	2004.155767	0.622256768
TCGA-BF-A5EO-01A-12R-A27Q-07	-461.2269363	1018.601353	557.3744169	0.773276889
TCGA-GN-A26A-06A-11R-A18T-07	284.1416237	1726.936183	2011.077807	0.62146105
TCGA-FS-A1ZN-01A-11R-A18T-07	-1092.18633	-206.0824632	-1298.268793	0.915351465

TCGA-FS-A4F0-06A-11R-A24X-07	-1543.435042	-491.330928	-2034.76597	0.953453606
TCGA-ER-A3EV-06A-11R-A20F-07	-560.1773929	681.4584641	121.2810712	0.812254287
TCGA-D3-A8GJ-06A-11R-A37K-07	-138.5971559	2636.616458	2498.019302	0.563923283
TCGA-ER-A198-06A-11R-A18T-07	-443.1186037	1241.52844	798.4098367	0.750363508
TCGA-YG-AA3P-06A-11R-A38C-07	-1262.531749	-433.5322261	-1696.063975	0.937289826
TCGA-EB-A44Q-06A-11R-A266-07	-445.9222041	2070.392337	1624.470133	0.664896881
TCGA-WE-AAA0-06A-11R-A38C-07	-879.1587861	1025.302862	146.144076	0.810120057
TCGA-D9-A4Z3-01A-11R-A266-07	-610.7643259	265.9385789	-344.8257469	0.850231834
TCGA-EB-A299-01A-21R-A18U-07	-279.0581493	694.953988	415.8958387	0.78627716
TCGA-YD-A9TB-06A-12R-A40A-07	382.1918192	881.9208203	1264.112639	0.703458585
TCGA-EB-A24D-01A-11R-A18T-07	-571.0121906	296.355557	-274.6566336	0.844764817
TCGA-D9-A4Z6-06A-12R-A266-07	-686.775038	-182.9305369	-869.7055749	0.888227231
TCGA-BF-A5ES-01A-11R-A27Q-07	-112.4547825	925.8299152	813.3751328	0.748909603
TCGA-ER-A197-06A-32R-A18S-07	312.347955	2167.039519	2479.387474	0.566179756
TCGA-EE-A3J8-06A-11R-A20F-07	-1353.360814	-854.6075223	-2207.968336	0.960811024
TCGA-WE-A8K6-06A-11R-A37K-07	-635.3824753	164.6361913	-470.746284	0.859816007
TCGA-W3-AA1O-06A-11R-A38C-07	-688.9308184	-129.776644	-818.7074625	0.884763306
TCGA-EE-A3AA-06A-11R-A18S-07	-423.1026223	1720.569103	1297.466481	0.699970434
TCGA-EE-A29V-06A-12R-A18S-07	-701.8255468	-240.2261569	-942.0517037	0.893055733
TCGA-D3-A2JA-06A-11R-A18T-07	985.4216634	1727.885503	2713.307166	0.53754847
TCGA-EE-A3JI-06A-11R-A21D-07	-1048.00892	332.3026154	-715.7063051	0.877616207
TCGA-W3-AA1Q-06A-11R-A38C-07	-542.3989966	1106.211441	563.8124448	0.772677331
TCGA-GN-A4U8-06A-11R-A32P-07	-482.512406	2096.866268	1614.353862	0.66600531
TCGA-D3-A8GO-06A-11R-A37K-07	-397.0363617	1472.202068	1075.165706	0.722897935
TCGA-D3-A3MU-06A-11R-A21D-07	220.1424298	852.6922228	1072.834653	0.723134318
TCGA-EE-A2A6-06A-11R-A18T-07	-678.4479277	1820.220271	1141.772343	0.71610797
TCGA-DA-A95X-06A-11R-A37K-07	-369.3615069	737.0508293	367.6893225	0.790629612
TCGA-ER-A19P-06A-11R-A18S-07	761.9512998	3362.210052	4124.161352	0.35267671
TCGA-BF-A3DN-01A-11R-A20F-07	105.3251096	-108.7688444	-3.443734819	0.822796776
TCGA-EB-A41B-01A-11R-A24X-07	-1337.257512	-43.58220527	-1380.839717	0.920164482
TCGA-D3-A5GU-06A-11R-A27Q-07	-336.4524025	1027.112186	690.6597834	0.760724299
TCGA-EB-A57M-01A-51R-A311-07	642.2239287	818.2802327	1460.504161	0.682680034
TCGA-GN-A263-01A-11R-A18T-07	268.078207	824.9012775	1092.979484	0.721088718
TCGA-ER-A19A-06A-21R-A18U-07	1730.22306	3209.235405	4939.458465	0.23843487
TCGA-D3-A3BZ-06A-12R-A18S-07	1356.352627	2149.317183	3505.669811	0.436061472
TCGA-DA-A1I2-06A-21R-A18U-07	-270.3837978	598.9894157	328.6056179	0.794129337
TCGA-EB-A4OZ-01A-12R-A266-07	-49.59519708	2140.098275	2090.503078	0.612285064
TCGA-GF-A769-01A-32R-A32P-07	-1067.161016	-173.1201087	-1240.281125	0.911890974
TCGA-EE-A2MN-06A-11R-A18S-07	-440.0445098	1690.472623	1250.428114	0.704884842
TCGA-GN-A26C-01A-11R-A18T-07	410.0907893	2493.068746	2903.159535	0.513843468
TCGA-D3-A2JK-06A-11R-A18S-07	32.32694159	922.6296961	954.9566376	0.734976869
TCGA-XV-AAZV-01A-11R-A40A-07	148.9508554	1943.890737	2092.841592	0.612013628
TCGA-FS-A1ZE-06A-11R-A18T-07	-664.4517924	-632.2565224	-1296.708315	0.915259209
TCGA-FS-A1ZM-06A-12R-A18S-07	713.5755324	1868.57125	2582.146783	0.553682456
TCGA-D3-A1Q7-06A-11R-A18T-07	978.6676363	2761.054114	3739.721751	0.404892585
TCGA-EE-A184-06A-11R-A18S-07	-762.5709206	1015.78865	253.2177299	0.800805966
TCGA-ER-A194-01A-11R-A18U-07	67.09402854	1525.392537	1592.486565	0.668396264
TCGA-DA-A1I1-06A-12R-A18U-07	404.8428215	1712.673737	2117.516558	0.609145173

TCGA-D3-A1QB-06A-11R-A18T-07	-122.9203724	2469.076105	2346.155733	0.582190834
TCGA-EE-A2MR-06A-11R-A18S-07	557.1996102	2870.254807	3427.454417	0.446364552
TCGA-D3-A2JD-06A-11R-A18T-07	25.50013341	1590.439726	1615.939859	0.665831631
TCGA-EE-A180-06A-11R-A21D-07	-555.0072737	-25.02710494	-580.0343786	0.867896244
TCGA-EE-A2MG-06A-11R-A18T-07	826.7581663	2737.58353	3564.341697	0.428294995
TCGA-GN-A266-06A-11R-A18T-07	1092.832352	2180.33302	3273.165372	0.466514798
TCGA-EB-A85I-01A-11R-A352-07	-390.3364061	1278.920687	888.5842805	0.741548326
TCGA-EB-A550-01A-61R-A27Q-07	-746.3360833	59.4900343	-686.846049	0.875577584
TCGA-Z2-A8RT-06A-11R-A37K-07	-374.4306885	1554.952438	1180.52175	0.712126268
TCGA-D3-A2JP-06A-11R-A18S-07	197.1143426	1636.847673	1833.962015	0.641617168
TCGA-D3-A51N-06A-11R-A266-07	-164.9640656	1623.500713	1458.536647	0.682891043
TCGA-ER-A19D-06A-11R-A18S-07	1142.120377	1413.165877	2555.286254	0.556961435
TCGA-GF-A6C8-06A-12R-A311-07	-423.2215757	1062.534431	639.3128554	0.765594811
TCGA-BF-A1PZ-01A-11R-A18S-07	-1063.842126	103.0045855	-960.8375405	0.894293082
TCGA-EE-A3AD-06A-11R-A18S-07	-1429.32854	-351.9797463	-1781.308287	0.941577712
TCGA-EB-A5KH-06A-11R-A27Q-07	-653.9043346	-134.524944	-788.4292786	0.882683263
TCGA-ER-A2NE-06A-21R-A18T-07	-1576.341232	-415.8040565	-1992.145289	0.95154846
TCGA-EB-A5UN-06A-11R-A311-07	-784.3844072	430.1897555	-354.1946517	0.850954971
TCGA-D3-A8GB-06A-11R-A37K-07	889.0106445	2425.930909	3314.941554	0.461081995
TCGA-D3-A2J7-06A-11R-A18T-07	203.0375097	1200.861048	1403.898558	0.688727963
TCGA-BF-AAP1-01A-11R-A39D-07	-1053.670708	-350.9282515	-1404.598959	0.921524383
TCGA-GF-A4EO-06A-12R-A24X-07	233.6614615	3036.442244	3270.103705	0.466912266
TCGA-D3-A5GR-06A-11R-A27Q-07	204.0383863	1925.495647	2129.534033	0.607745249
TCGA-EE-A2GN-06A-11R-A18S-07	-104.0193235	72.16434379	-31.85497969	0.825159824
TCGA-FR-A8YE-06A-11R-A37K-07	572.3892015	2944.184707	3516.573909	0.434620512
TCGA-GN-A4U3-06A-11R-A32P-07	-1095.598383	-28.12990853	-1123.728291	0.904735935
TCGA-EE-A2A1-06A-11R-A18T-07	921.4768497	2149.904564	3071.381414	0.492505225
TCGA-EE-A2MF-06A-11R-A21D-07	-811.0918498	711.3821139	-99.70973595	0.830745362
TCGA-EE-A2M8-06A-12R-A18S-07	1139.586163	3200.097687	4339.68385	0.322901749
TCGA-EE-A3J3-06A-11R-A20F-07	446.8936176	894.9357526	1341.82937	0.695304969
TCGA-EB-A430-01A-11R-A24X-07	-1146.586157	479.1347751	-667.4513817	0.874198758
TCGA-D3-A51F-06A-11R-A266-07	1201.732948	3404.644394	4606.377341	0.285613339
TCGA-EB-A431-01A-11R-A266-07	-1112.729233	-78.07433152	-1190.803564	0.908886089
TCGA-GN-A9SD-06A-11R-A40A-07	249.501823	2319.561524	2569.063347	0.555280685
TCGA-FW-A5DX-01A-11R-A27Q-07	-998.9046082	-376.5341183	-1375.438726	0.919853786
TCGA-EB-A44P-01A-11R-A266-07	-763.5717359	214.9272136	-548.6445222	0.865598201
TCGA-EB-A5SH-06A-11R-A311-07	-680.3106902	613.3275533	-66.98313694	0.828061692
TCGA-ER-A199-06A-11R-A18T-07	-175.4921524	2469.558169	2294.066017	0.588390487
TCGA-FW-A5DY-06A-11R-A311-07	-82.34718815	2627.820088	2545.472899	0.558157236
TCGA-WE-A8ZX-06A-11R-A37K-07	-470.4521311	1668.229869	1197.777738	0.710345704
TCGA-EE-A3J4-06A-11R-A20F-07	-437.6594061	485.6443285	47.98492234	0.818482931
TCGA-D3-A1Q8-06A-11R-A18T-07	925.8130213	1175.421908	2101.234929	0.611038805
TCGA-EE-A29M-06A-11R-A18T-07	-887.1766398	1299.627362	412.450722	0.78658952
TCGA-DA-A3F8-06A-11R-A20F-07	257.7186297	2988.298437	3246.017067	0.47003592
TCGA-XV-AAZY-01A-12R-A40A-07	-133.3161999	1431.280033	1297.963833	0.699918293
TCGA-EE-A17Z-06A-11R-A18S-07	-1025.410203	-742.0215426	-1767.431746	0.940889734
TCGA-EE-A29E-06A-11R-A18T-07	-1227.865414	-201.1895822	-1429.054997	0.92291246
TCGA-D9-A4Z5-01A-11R-A266-07	-161.6870649	363.1410949	201.4540299	0.805333615

TCGA-FR-A2OS-01A-11R-A21D-07	-570.334212	-2.165431869	-572.4996439	0.867346307
TCGA-YD-A9TA-06A-11R-A39D-07	6.225019618	1230.000488	1236.225507	0.706362087
TCGA-W3-A825-06A-11R-A352-07	128.9723504	1555.029324	1684.001674	0.658344455
TCGA-DA-A3F2-06A-11R-A20F-07	1832.792009	3265.350922	5098.142931	0.215751003
TCGA-FR-A8YD-06A-11R-A37K-07	-1040.367963	259.7600407	-780.6079226	0.882143118
TCGA-BF-A1PX-01A-12R-A18T-07	494.483791	2195.827807	2690.311598	0.54039172
TCGA-FS-A4FC-06A-11R-A24X-07	-276.3342306	454.0004826	177.666252	0.807398698
TCGA-D3-A5GT-01A-12R-A311-07	-1214.126041	-482.4417379	-1696.567778	0.9373156
TCGA-D3-A5GL-06A-11R-A27Q-07	-919.8583669	-138.6784038	-1058.536771	0.900618286
TCGA-DA-A3F5-06A-11R-A20F-07	-678.1957713	332.3929147	-345.8028566	0.850307327
TCGA-EE-A2MT-06A-11R-A18S-07	-1309.490809	144.8516492	-1164.63916	0.907277676
TCGA-EE-A20I-06A-11R-A18U-07	35.94932243	638.6886837	674.6380061	0.762248696
TCGA-GF-A6C9-06A-11R-A311-07	416.9253955	2686.04799	3102.973386	0.488464027
TCGA-WE-A8ZN-06A-11R-A37K-07	530.1957338	1876.303684	2406.499418	0.574966273
TCGA-D3-A51G-06A-11R-A266-07	-40.66634067	395.3232611	354.6569205	0.791799487
TCGA-FS-A1ZP-06A-11R-A18T-07	447.285572	996.802829	1444.088401	0.684438819
TCGA-ER-A19F-06A-11R-A18S-07	-740.312814	248.4122484	-491.9005655	0.861397388
TCGA-FR-A3YN-06A-11R-A239-07	-690.9015966	915.3339119	224.4323153	0.803329481
TCGA-EB-A44N-01A-11R-A266-07	-126.4475888	1107.691277	981.2436884	0.732354894
TCGA-IH-A3EA-01A-11R-A20F-07	-1061.596225	658.252042	-403.3441831	0.854722169
TCGA-EE-A2GR-06A-11R-A18S-07	-1029.534452	-731.7705323	-1761.304984	0.940584737
TCGA-GN-A267-06A-21R-A18T-07	-325.1184123	970.7928332	645.6744209	0.76499374
TCGA-EE-A183-06A-11R-A18S-07	-6.634563667	1953.070083	1946.43552	0.628866867
TCGA-D3-A2JL-06A-11R-A18S-07	-511.7135572	1873.664338	1361.950781	0.69317915
TCGA-EE-A20C-06A-11R-A18S-07	-1511.881039	-176.7747719	-1688.655811	0.936910246
TCGA-RP-A6K9-06A-41R-A352-07	51.8126037	1242.857455	1294.670059	0.70026353
TCGA-D3-A8GQ-06A-11R-A37K-07	-558.5952934	97.97763111	-460.6176623	0.859055909
TCGA-EB-A551-01A-21R-A27Q-07	274.4687849	2299.683102	2574.151887	0.554659329
TCGA-GN-A8LK-06A-11R-A37K-07	-1726.227119	-840.1733562	-2566.400476	0.974059479
TCGA-GN-A26D-06A-11R-A18T-07	-1042.896291	127.3329833	-915.5633078	0.891299512
TCGA-FS-A1ZK-06A-11R-A18T-07	-1034.038815	-800.306326	-1834.345141	0.944171172
TCGA-FW-A3R5-06A-11R-A239-07	-140.3092507	743.084285	602.7750343	0.769034136
TCGA-D3-A8GP-06A-11R-A37K-07	-1290.743902	-309.7208548	-1600.464757	0.932306536
TCGA-EE-A2MS-06A-11R-A18S-07	-538.2765839	638.7058421	100.4292582	0.814035857
TCGA-D9-A6EG-06A-12R-A32P-07	-513.5491506	-424.8829766	-938.4321271	0.892816545
TCGA-BF-AAP2-01A-11R-A40A-07	-917.0991561	713.3236022	-203.7755539	0.839151342
TCGA-DA-A1IA-06A-11R-A18S-07	-1311.342172	-22.06038159	-1333.402553	0.917415871
TCGA-GN-A262-06A-11R-A18T-07	-1597.511795	-1010.869135	-2608.38093	0.975435443
TCGA-EE-A2MC-06A-12R-A18S-07	530.3353655	2414.377706	2944.713071	0.508601212
TCGA-EE-A3AC-06A-11R-A18S-07	-992.3166132	1402.026568	409.7099546	0.786837875
TCGA-GN-A8LN-01A-11R-A37K-07	-668.5015789	409.7485942	-258.7529848	0.843513252
TCGA-D9-A6E9-06A-12R-A311-07	175.4439839	2796.847463	2972.291447	0.505111557
TCGA-EB-A6QY-01A-12R-A32P-07	-1108.144377	-48.56041894	-1156.704796	0.906787279
TCGA-FR-A69P-06A-21R-A311-07	99.65030366	-93.20245853	6.447845132	0.821970703
TCGA-D3-A2JB-06A-11R-A18T-07	-183.483133	2737.356152	2553.873019	0.557133715
TCGA-EE-A2A0-06A-11R-A18T-07	1901.986035	2080.906859	3982.892894	0.372003643
TCGA-EB-A24C-01A-11R-A18T-07	-433.1686432	-297.4899716	-730.6586148	0.878666212
TCGA-ER-A19H-06A-12R-A18S-07	233.7680446	1163.576495	1397.344539	0.689425153

TCGA-FS-A4FD-06A-11R-A266-07	30.53788872	63.47127755	94.00916627	0.814582852
TCGA-D3-A51R-06A-11R-A266-07	-880.0590946	-1476.277672	-2356.336767	0.966619858
TCGA-D3-A3CF-06A-11R-A18T-07	372.2679804	2243.48442	2615.7524	0.54956796
TCGA-EE-A3JE-06A-11R-A20F-07	1533.653565	2901.335759	4434.989324	0.30963024
TCGA-D3-A8GN-06A-11R-A37K-07	-166.368043	1884.122104	1717.754061	0.654607086
TCGA-FS-A1ZU-06A-12R-A18T-07	-494.9125628	-280.5091774	-775.4217402	0.881784318
TCGA-GN-A4U9-06A-11R-A32P-07	-683.1870547	84.75262277	-598.4344319	0.869234743
TCGA-D3-A1Q1-06A-21R-A18T-07	-889.9613239	-621.9768072	-1511.938131	0.92752818
TCGA-EE-A2M7-06A-11R-A18U-07	-50.05660329	1077.103523	1027.046919	0.727760269
TCGA-EE-A3JH-06A-11R-A21D-07	874.328278	3129.152159	4003.480437	0.369196822
TCGA-D3-A8GR-06A-11R-A37K-07	-998.2720192	-25.80754563	-1024.079565	0.898408549
TCGA-EB-A85J-01A-12R-A352-07	-839.4837231	2007.967766	1168.484043	0.713365678
TCGA-XV-AAZW-01A-12R-A40A-07	-591.8783698	-320.6801634	-912.5585331	0.891099439
TCGA-EE-A29H-06A-12R-A18S-07	-562.0446363	1003.972131	441.9274945	0.783910439
TCGA-EE-A2M6-06A-12R-A18S-07	-694.3593137	1147.91805	453.5587364	0.78284926
TCGA-RP-A695-06A-11R-A311-07	-896.2224822	483.1136164	-413.1088657	0.855465318
TCGA-D3-A2JH-06A-11R-A18T-07	8.816752109	2401.539061	2410.355813	0.574503033
TCGA-FS-A1YW-06A-11R-A18T-07	-727.7797296	195.3018245	-532.477905	0.864407475
TCGA-EE-A2ML-06A-11R-A18S-07	-481.2001929	1304.282882	823.082689	0.747964564
TCGA-D3-A1Q9-06A-11R-A18T-07	-386.456447	725.5436901	339.0872431	0.793193327
TCGA-EE-A17X-06A-11R-A18S-07	-1030.052415	229.1688379	-800.8835774	0.883540959
TCGA-D3-A1Q5-06A-11R-A18T-07	-306.3089508	-813.910189	-1120.21914	0.904516395
TCGA-D3-A2JG-06A-11R-A18T-07	-796.2813652	151.665251	-644.6161142	0.872566248
TCGA-GN-A4U5-01A-11R-A32P-07	-252.0312667	1844.963907	1592.93264	0.668347559
TCGA-EE-A2GE-06A-11R-A18T-07	1401.945014	2537.980291	3939.925305	0.377850693
TCGA-DA-A1I4-06A-11R-A18U-07	840.9495617	1593.66151	2434.611072	0.571585209
TCGA-BF-AAOU-01A-12R-A39D-07	-331.2356868	670.726547	339.4908603	0.793157246
TCGA-FR-A44A-06A-11R-A24X-07	412.4890479	2859.540407	3272.029454	0.466662275
TCGA-D9-A6EA-06A-11R-A311-07	-1278.94207	-36.41414197	-1315.356212	0.91635854
TCGA-EB-A5UL-06A-11R-A311-07	-873.121887	1366.920666	493.7987795	0.779160364
TCGA-FS-A1ZY-06A-11R-A18S-07	-821.3142322	-763.4870217	-1584.801254	0.931472516
TCGA-WE-A8K5-06A-11R-A37K-07	114.99508	784.5933687	899.5884487	0.740463666
TCGA-FS-A4F4-06A-12R-A266-07	482.8709001	1276.713905	1759.584806	0.649952921
TCGA-DA-A1IC-06A-11R-A18S-07	-289.1674145	522.6622315	233.494817	0.802536549
TCGA-YD-A89C-06A-11R-A37K-07	-952.9267649	-692.2263714	-1645.153136	0.934658914
TCGA-EB-A41A-01A-11R-A24X-07	-1145.320038	-32.57813464	-1177.898172	0.908094426
TCGA-D3-A3MR-06A-11R-A21D-07	244.1572293	2490.323337	2734.480566	0.534925102
TCGA-ER-A3ES-06A-11R-A20F-07	-1328.458017	-1081.603958	-2410.061974	0.96861035
TCGA-RP-A690-06A-11R-A311-07	-1056.109375	-48.98600633	-1105.095382	0.903567476
TCGA-D3-A8GK-06A-11R-A37K-07	-1590.58541	-725.2272818	-2315.812692	0.965078681
TCGA-EE-A2GU-06A-11R-A18T-07	-961.2167328	1641.876163	680.6594303	0.76167628
TCGA-FS-A1ZC-06A-11R-A18T-07	-675.1160892	-620.2981794	-1295.414269	0.915182668
TCGA-3N-A9WD-06A-11R-A38C-07	-520.9028824	1427.705383	906.8025006	0.739751541
TCGA-D3-A8GL-06A-11R-A37K-07	-1255.03784	-951.8248084	-2206.862649	0.960766021
TCGA-FR-A3R1-01A-11R-A239-07	-658.9287617	650.8570719	-8.071689845	0.823182673
TCGA-ER-A19T-01A-11R-A18T-07	-746.9928467	-1027.403879	-1774.396725	0.941235535
TCGA-HR-A2OG-06A-21R-A18U-07	996.1902818	1564.193498	2560.38378	0.556339823
TCGA-EB-A1NK-01A-11R-A18T-07	-188.55283	525.5724625	337.0196325	0.793378113

TCGA-D9-A1X3-06A-11R-A18S-07	-827.175924	-690.2807977	-1517.456722	0.92783064
TCGA-GN-A268-06A-11R-A18T-07	-1282.909387	-460.5634224	-1743.47281	0.9396927
TCGA-YG-AA3N-01A-11R-A38C-07	-487.9703066	1912.988514	1425.018208	0.686477009
TCGA-EE-A3JA-06A-11R-A20F-07	198.6286333	1318.016714	1516.645347	0.676635171
TCGA-D3-A3MV-06A-11R-A21D-07	-585.1639342	-17.2054119	-602.3693461	0.869520164
TCGA-D3-A2J6-06A-11R-A18T-07	-617.2777132	946.7004374	329.4227242	0.794056437
TCGA-D3-A8GV-06A-11R-A37K-07	-870.0628268	2.027999015	-868.0348277	0.888114537
TCGA-EB-A3Y7-01A-11R-A239-07	-907.4261142	97.60630064	-809.8198136	0.884154555
TCGA-EE-A2GI-06A-11R-A18T-07	-408.012673	1635.75712	1227.744447	0.707242758
TCGA-EB-A5VU-01A-21R-A32P-07	-441.6151904	-27.26640393	-468.8815943	0.859676215
TCGA-D9-A3Z3-06A-11R-A239-07	196.7922084	1635.596507	1832.388716	0.641794289
TCGA-BF-A9VF-01A-11R-A37K-07	-1355.736335	-395.2749144	-1751.011249	0.940070589
TCGA-D3-A51K-06A-11R-A266-07	-710.3973122	1244.118104	533.7207915	0.775473759
TCGA-EE-A29N-06A-12R-A18S-07	-111.5020669	1986.021288	1874.519221	0.637039474
TCGA-ER-A193-06A-12R-A18S-07	581.5925438	1755.055704	2336.648248	0.583324953
TCGA-BF-A3DJ-01A-11R-A20F-07	-77.25834511	1200.277294	1123.018949	0.718026666
TCGA-DA-A960-01A-11R-A37K-07	-1388.679434	-173.2448461	-1561.92428	0.930245559
TCGA-EB-A3XB-01A-11R-A239-07	-543.8327704	742.3003497	198.4675792	0.805593416
TCGA-D3-A51T-06A-11R-A266-07	-845.4362092	874.6857298	29.24952057	0.820059874
TCGA-D9-A149-06A-11R-A18S-07	-231.7280007	1395.666303	1163.938302	0.713833131
TCGA-GN-A4U4-06A-11R-A32P-07	-1294.773003	-65.95155342	-1360.724557	0.919004407
TCGA-DA-A95Z-06A-11R-A37K-07	-1071.609751	195.4805923	-876.1291587	0.888660012
TCGA-FR-A7U8-06A-21R-A352-07	-1106.458503	-350.0739081	-1456.532411	0.924457834
TCGA-FS-A1ZW-06A-12R-A18T-07	-174.5982414	1872.387708	1697.789466	0.656819693
TCGA-D3-A8GE-06A-11R-A37K-07	1004.557645	1556.089243	2560.646888	0.55630773
TCGA-FS-A1ZT-06A-11R-A18U-07	-103.6504574	1574.322501	1470.672043	0.681588659
TCGA-EE-A2GL-06A-11R-A18S-07	-267.4611635	2439.090222	2171.629058	0.602826688
TCGA-ER-A19Q-06A-11R-A18U-07	550.3234828	1991.631155	2541.954638	0.55858567
TCGA-FW-A3TU-06A-11R-A239-07	-1328.558379	945.5927245	-382.9656545	0.85316559
TCGA-EB-A5UM-01A-11R-A311-07	-726.7527732	1425.711184	698.9584105	0.759933069
TCGA-BF-A5EP-01A-12R-A27Q-07	-729.3075127	-264.3726335	-993.6801462	0.896439952
TCGA-D3-A2J9-06A-11R-A18T-07	465.5301165	3231.715177	3697.245294	0.410585786
TCGA-EE-A3AB-06A-11R-A18S-07	300.2832949	1700.69702	2000.980315	0.622621585
TCGA-EE-A2GC-06A-11R-A18T-07	184.7342261	1770.43526	1955.169486	0.627869544
TCGA-D3-A8GI-06A-11R-A37K-07	241.0121337	427.2244188	668.2365525	0.762856586
TCGA-D3-A8GC-06A-11R-A37K-07	-945.700216	456.5133855	-489.1868306	0.861194988
TCGA-Z2-AA3V-06A-11R-A39D-07	124.5579745	2436.329893	2560.887867	0.556278335
TCGA-FS-A1Z4-06A-11R-A18T-07	270.9298779	2418.713303	2689.643181	0.540474274
TCGA-BF-A3DL-01A-11R-A20F-07	-331.1286601	145.38416	-185.7445002	0.837708842
TCGA-XV-AB01-06A-12R-A40A-07	109.3626051	1070.232843	1179.595448	0.71222172
TCGA-FS-A1ZB-06A-12R-A18S-07	824.5279672	1505.973622	2330.501589	0.584057564
