# Supplementary material for: Hierarchical Clustering of Cutaneous Melanoma Based on Immunogenomic Profiling
Source: Front Oncol. 2020 Nov 30;10:580029. doi: 10.3389/fonc.2020.580029 (PMC7735560; doi:10.3389/fonc.2020.580029)
Supplement: Supplementary Table 2 — GO terms enriched in Immunity_H and Immunity_L. GO, gene oncology. [file DataSheet_2.pdf]

Table S2 GO terms enriched in Immunity_H and Immunity_L. GO, gene oncology

ONTOLOGY	ID	Description	setSize	enrichmentScore	NES	pvalue	p.adjust	qvalues	rank	leading_edge
CC	GO:0019814	immunoglobulin complex	142	0.8787284	1.819717	0.000999	0.049713	0.049137	3333	tags=88%, list=9%, signal=80%
CC	GO:0042571	immunoglobulin complex, circulating	61	0.8563384	1.750517	0.001007	0.049713	0.049137	3333	tags=87%, list=9%, signal=79%
MF	GO:0034987	immunoglobulin receptor binding	65	0.8438765	1.727776	0.001005	0.049713	0.049137	5545	tags=92%, list=15%, signal=78%
BP	GO:0006958	complement activation, classical pathway	126	0.8347035	1.726125	0.000999	0.049713	0.049137	3333	tags=69%, list=9%, signal=63%
BP	GO:0006910	phagocytosis, recognition	73	0.8223429	1.688376	0.001003	0.049713	0.049137	3333	tags=70%, list=9%, signal=64%
CC	GO:0042101	T cell receptor complex	119	0.8203211	1.695757	0.000999	0.049713	0.049137	5939	tags=87%, list=16%, signal=73%
BP	GO:0002455	humoral immune response mediated by circulating immunoglobulin	139	0.813442	1.684871	0.000999	0.049713	0.049137	3333	tags=63%, list=9%, signal=58%
MF	GO:0003823	antigen binding	147	0.8068972	1.671196	0.000999	0.049713	0.049137	6114	tags=82%, list=17%, signal=68%
CC	GO:0045095	keratin filament	94	0.8005899	1.649673	0.001001	0.049713	0.049137	5963	tags=76%, list=16%, signal=63%
BP	GO:0002433	immune response-regulating cell surface receptor signaling pathway involved in phagocytosis	138	0.8003111	1.6572	0.000999	0.049713	0.049137	2263	tags=40%, list=6%, signal=38%
BP	GO:0038096	Fc-gamma receptor signaling pathway involved in phagocytosis	138	0.8003111	1.6572	0.000999	0.049713	0.049137	2263	tags=40%, list=6%, signal=38%
BP	GO:0038094	Fc-gamma receptor signaling pathway	141	0.7984798	1.653273	0.000999	0.049713	0.049137	5805	tags=52%, list=16%, signal=44%
BP	GO:0002431	Fc receptor mediated stimulatory signaling pathway	144	0.7938699	1.643615	0.000999	0.049713	0.049137	5805	tags=49%, list=16%, signal=42%
BP	GO:0050853	B cell receptor signaling pathway	114	0.7902847	1.634695	0.000999	0.049713	0.049137	5960	tags=68%, list=16%, signal=57%
CC	GO:0001533	cornified envelope	65	0.7877634	1.612889	0.001005	0.049713	0.049137	6577	tags=80%, list=18%, signal=66%
BP	GO:0006911	phagocytosis, engulfment	107	0.7867859	1.626365	0.000999	0.049713	0.049137	6069	tags=64%, list=17%, signal=53%
BP	GO:0006956	complement activation	164	0.785075	1.626428	0.000999	0.049713	0.049137	6175	tags=69%, list=17%, signal=57%
BP	GO:0038095	Fc-epsilon receptor signaling pathway	167	0.7846856	1.624472	0.000999	0.049713	0.049137	2263	tags=31%, list=6%, signal=29%
BP	GO:0099024	plasma membrane invagination	116	0.7836281	1.619192	0.000999	0.049713	0.049137	6069	tags=59%, list=17%, signal=49%

BP	GO:0010324	membrane invagination	124	0.7819539	1.617021	0.000999	0.049713	0.049137	6069	tags=55%, list=17%, signal=46%
BP	GO:0030449	regulation of complement activation	114	0.7818017	1.617147	0.000999	0.049713	0.049137	2284	tags=51%, list=6%, signal=48%
BP	GO:0016064	immunoglobulin mediated immune response	207	0.7808383	1.622483	0.000999	0.049713	0.049137	6074	tags=58%, list=17%, signal=49%
BP	GO:2000257	regulation of protein activation cascade	115	0.7797706	1.612746	0.000999	0.049713	0.049137	2284	tags=50%, list=6%, signal=47%
BP	GO:0019724	B cell mediated immunity	210	0.77918	1.618892	0.000999	0.049713	0.049137	6074	tags=59%, list=17%, signal=49%
BP	GO:0050871	positive regulation of B cell activation	131	0.7789443	1.612268	0.000999	0.049713	0.049137	6234	tags=56%, list=17%, signal=47%
BP	GO:0002377	immunoglobulin production	192	0.7748414	1.607422	0.000999	0.049713	0.049137	5412	tags=47%, list=15%, signal=41%
BP	GO:0002920	regulation of humoral immune response	133	0.7744267	1.602514	0.000999	0.049713	0.049137	6175	tags=66%, list=17%, signal=55%
BP	GO:0031424	keratinization	223	0.7717372	1.604348	0.000999	0.049713	0.049137	6496	tags=74%, list=18%, signal=62%
BP	GO:0070268	cornification	112	0.7662564	1.58416	0.000999	0.049713	0.049137	6496	tags=74%, list=18%, signal=61%
BP	GO:0072376	protein activation cascade	187	0.7632783	1.583517	0.000999	0.049713	0.049137	6175	tags=61%, list=17%, signal=51%
BP	GO:0018149	peptide cross-linking	60	0.759579	1.550782	0.001007	0.049713	0.049137	6577	tags=68%, list=18%, signal=56%
BP	GO:0050864	regulation of B cell activation	173	0.7548637	1.563726	0.000999	0.049713	0.049137	5960	tags=49%, list=16%, signal=41%
MF	GO:0008009	chemokine activity	47	0.7506953	1.525151	0.0010132	0.049713	0.049137	7910	tags=79%, list=22%, signal=62%
BP	GO:0030216	keratinocyte differentiation	301	0.7495571	1.565698	0.000999	0.049713	0.049137	6496	tags=60%, list=18%, signal=50%
BP	GO:0002449	lymphocyte mediated immunity	341	0.7494887	1.567848	0.000999	0.049713	0.049137	6621	tags=54%, list=18%, signal=45%
BP	GO:0038093	Fc receptor signaling pathway	239	0.7481168	1.557637	0.000999	0.049713	0.049137	6159	tags=35%, list=17%, signal=29%
CC	GO:0005882	intermediate filament	204	0.7466599	1.550021	0.000999	0.049713	0.049137	6080	tags=56%, list=17%, signal=47%
BP	GO:0006959	humoral immune response	336	0.745538	1.559968	0.000999	0.049713	0.049137	6175	tags=57%, list=17%, signal=48%
BP	GO:0002673	regulation of acute inflammatory response	156	0.7451872	1.543005	0.000999	0.049713	0.049137	6207	tags=53%, list=17%, signal=44%

BP	GO:0002460	adaptive immune response based on somatic recombination of immune receptors built from immunoglobulin superfamily domains	349	0.7409429	1.549714	0.000999	0.049713	0.049137	7587	tags=57%, list=21%, signal=46%
BP	GO:0002440	production of molecular mediator of immune response	284	0.7382235	1.540338	0.000999	0.049713	0.049137	6684	tags=46%, list=18%, signal=38%
BP	GO:0031640	killing of cells of other organism	60	0.7319152	1.494302	0.001007	0.049713	0.049137	6419	tags=52%, list=18%, signal=43%
BP	GO:0044364	disruption of cells of other organism	60	0.7319152	1.494302	0.001007	0.049713	0.049137	6419	tags=52%, list=18%, signal=43%
BP	GO:0070613	regulation of protein processing	179	0.7306872	1.51504	0.000999	0.049713	0.049137	6194	tags=45%, list=17%, signal=38%
BP	GO:0009913	epidermal cell differentiation	354	0.7298283	1.527009	0.000999	0.049713	0.049137	6496	tags=53%, list=18%, signal=44%
MF	GO:0042379	chemokine receptor binding	63	0.7290182	1.491394	0.001006	0.049713	0.049137	7602	tags=70%, list=21%, signal=55%
CC	GO:0045111	intermediate filament cytoskeleton	241	0.7286016	1.517827	0.000999	0.049713	0.049137	7414	tags=56%, list=20%, signal=45%
BP	GO:1903317	regulation of protein maturation	181	0.7279973	1.509403	0.000999	0.049713	0.049137	6194	tags=45%, list=17%, signal=37%
BP	GO:0002709	regulation of T cell mediated immunity	70	0.7244727	1.484581	0.001004	0.049713	0.049137	7850	tags=59%, list=22%, signal=46%
BP	GO:0002429	immune response-activating cell surface receptor signaling pathway	455	0.7240028	1.515692	0.000999	0.049713	0.049137	6824	tags=42%, list=19%, signal=34%
BP	GO:0009620	response to fungus	52	0.7228088	1.477872	0.0010081	0.049713	0.049137	7162	tags=46%, list=20%, signal=37%
BP	GO:0050851	antigen receptor-mediated signaling pathway	299	0.7215192	1.50699	0.000999	0.049713	0.049137	6000	tags=38%, list=16%, signal=32%
BP	GO:0006909	phagocytosis	355	0.7213579	1.50922	0.000999	0.049713	0.049137	6136	tags=39%, list=17%, signal=33%
BP	GO:0045058	T cell selection	47	0.7202281	1.463252	0.0010132	0.049713	0.049137	7984	tags=62%, list=22%, signal=48%
BP	GO:0002768	immune response-regulating cell surface receptor signaling pathway	488	0.7199692	1.508229	0.000999	0.049713	0.049137	6824	tags=41%, list=19%, signal=34%
BP	GO:0061844	antimicrobial humoral immune response mediated by antimicrobial peptide	64	0.7175537	1.468061	0.001006	0.049713	0.049137	6684	tags=52%, list=18%, signal=42%
BP	GO:0070098	chemokine-mediated signaling pathway	86	0.7172049	1.475164	0.001	0.049713	0.049137	7518	tags=66%, list=21%, signal=53%

BP	GO:0048247	lymphocyte chemotaxis	62	0.7138958	1.458596	0.001006	0.049713	0.049137	7914	tags=61%, list=22%, signal=48%
BP	GO:0042113	B cell activation	298	0.7110573	1.484976	0.000999	0.049713	0.049137	7651	tags=47%, list=21%, signal=37%
BP	GO:0051251	positive regulation of lymphocyte activation	322	0.7083333	1.481723	0.000999	0.049713	0.049137	7890	tags=51%, list=22%, signal=41%
BP	GO:0043588	skin development	415	0.706598	1.478364	0.000999	0.049713	0.049137	6496	tags=47%, list=18%, signal=39%
BP	GO:1990868	response to chemokine	95	0.7044158	1.453444	0.001	0.049713	0.049137	7518	tags=63%, list=21%, signal=50%
BP	GO:1990869	cellular response to chemokine	95	0.7044158	1.453444	0.001	0.049713	0.049137	7518	tags=63%, list=21%, signal=50%
BP	GO:0072678	T cell migration	65	0.7032005	1.439752	0.001005	0.049713	0.049137	9232	tags=63%, list=25%, signal=47%
BP	GO:0002696	positive regulation of leukocyte activation	365	0.7018282	1.467992	0.000999	0.049713	0.049137	7890	tags=51%, list=22%, signal=40%
BP	GO:0031343	positive regulation of cell killing	68	0.7015694	1.43545	0.001005	0.049713	0.049137	8066	tags=63%, list=22%, signal=49%
BP	GO:0002526	acute inflammatory response	217	0.7015037	1.457922	0.000999	0.049713	0.049137	6824	tags=46%, list=19%, signal=37%
BP	GO:0050867	positive regulation of cell activation	376	0.7014032	1.467907	0.000999	0.049713	0.049137	7890	tags=51%, list=22%, signal=40%
BP	GO:0008544	epidermis development	459	0.6989568	1.463271	0.000999	0.049713	0.049137	6496	tags=44%, list=18%, signal=37%
BP	GO:0042102	positive regulation of T cell proliferation	94	0.6974539	1.437154	0.001001	0.049713	0.049137	7958	tags=60%, list=22%, signal=47%
BP	GO:0042742	defense response to bacterium	309	0.6957388	1.453543	0.000999	0.049713	0.049137	7579	tags=54%, list=21%, signal=43%
BP	GO:0030593	neutrophil chemotaxis	100	0.6952311	1.434344	0.001	0.049713	0.049137	9232	tags=72%, list=25%, signal=54%
BP	GO:1990266	neutrophil migration	114	0.6925289	1.432488	0.000999	0.049713	0.049137	9232	tags=71%, list=25%, signal=53%
BP	GO:0019730	antimicrobial humoral response	113	0.6920994	1.430355	0.000999	0.049713	0.049137	7656	tags=57%, list=21%, signal=45%
BP	GO:0001906	cell killing	166	0.6920956	1.433206	0.000999	0.049713	0.049137	8066	tags=55%, list=22%, signal=43%
CC	GO:0098802	plasma membrane receptor complex	287	0.6912342	1.442005	0.000999	0.049713	0.049137	6563	tags=45%, list=18%, signal=37%
BP	GO:0008037	cell recognition	204	0.6903714	1.433169	0.000999	0.049713	0.049137	6218	tags=38%, list=17%, signal=31%
BP	GO:0002697	regulation of immune effector process	453	0.6863314	1.437126	0.000999	0.049713	0.049137	8131	tags=49%, list=22%, signal=38%

BP	GO:0002456	T cell mediated immunity	106	0.6856048	1.417209	0.000999	0.049713	0.049137	8466	tags=55%, list=23%, signal=42%
BP	GO:0031341	regulation of cell killing	98	0.6845063	1.413547	0.000999	0.049713	0.049137	8066	tags=58%, list=22%, signal=45%
BP	GO:0051249	regulation of lymphocyte activation	472	0.6839544	1.432451	0.000999	0.049713	0.049137	7890	tags=47%, list=22%, signal=37%
BP	GO:0001910	regulation of leukocyte mediated cytotoxicity	78	0.6824026	1.404283	0.001	0.049713	0.049137	8998	tags=65%, list=25%, signal=49%
BP	GO:0072676	lymphocyte migration	109	0.6821408	1.410333	0.000999	0.049713	0.049137	8298	tags=55%, list=23%, signal=43%
BP	GO:0071621	granulocyte chemotaxis	118	0.6797813	1.404966	0.000999	0.049713	0.049137	8226	tags=59%, list=23%, signal=46%
BP	GO:0032649	regulation of interferon-gamma production	99	0.6786504	1.401547	0.000999	0.049713	0.049137	8364	tags=61%, list=23%, signal=47%
BP	GO:0046632	alpha-beta T cell differentiation	100	0.6778671	1.39852	0.001	0.049713	0.049137	8172	tags=50%, list=22%, signal=39%
BP	GO:0097530	granulocyte migration	136	0.677836	1.402856	0.000999	0.049713	0.049137	9232	tags=66%, list=25%, signal=50%
BP	GO:0060333	interferon-gamma-mediated signaling pathway	89	0.6768161	1.393453	0.001001	0.049713	0.049137	8629	tags=57%, list=24%, signal=44%
BP	GO:0071674	mononuclear cell migration	85	0.6767783	1.392574	0.001	0.049713	0.049137	8125	tags=56%, list=22%, signal=44%
CC	GO:0009897	external side of plasma membrane	382	0.6762608	1.415227	0.000999	0.049713	0.049137	6894	tags=46%, list=19%, signal=37%
BP	GO:0042129	regulation of T cell proliferation	154	0.6756235	1.399318	0.000999	0.049713	0.049137	8186	tags=55%, list=22%, signal=42%
BP	GO:0001909	leukocyte mediated cytotoxicity	107	0.6736761	1.392556	0.000999	0.049713	0.049137	8998	tags=61%, list=25%, signal=46%
BP	GO:0002286	T cell activation involved in immune response	105	0.6731855	1.390988	0.000999	0.049713	0.049137	8435	tags=57%, list=23%, signal=44%
BP	GO:0046631	alpha-beta T cell activation	137	0.6704276	1.387396	0.000999	0.049713	0.049137	8438	tags=52%, list=23%, signal=40%
BP	GO:0050900	leukocyte migration	488	0.6696506	1.402819	0.000999	0.049713	0.049137	7914	tags=46%, list=22%, signal=37%
MF	GO:0005549	odorant binding	98	0.6675157	1.37846	0.000999	0.049713	0.049137	6771	tags=54%, list=19%, signal=44%
BP	GO:0042100	B cell proliferation	95	0.6642501	1.370569	0.001	0.049713	0.049137	6544	tags=42%, list=18%, signal=35%

BP	GO:0002824	positive regulation of adaptive immune response based on somatic recombination of immune receptors built from immunoglobulin superfamily domains	99	0.6641226	1.371544	0.000999	0.049713	0.049137	7850	tags=47%, list=22%, signal=37%
BP	GO:0002708	positive regulation of lymphocyte mediated immunity	105	0.6636073	1.371197	0.000999	0.049713	0.049137	8066	tags=50%, list=22%, signal=39%
BP	GO:0032609	interferon-gamma production	111	0.6626915	1.370387	0.000999	0.049713	0.049137	8364	tags=59%, list=23%, signal=45%
BP	GO:0002706	regulation of lymphocyte mediated immunity	149	0.6625163	1.372261	0.000999	0.049713	0.049137	8066	tags=49%, list=22%, signal=38%
BP	GO:0002821	positive regulation of adaptive immune response	104	0.6599016	1.362895	0.000999	0.049713	0.049137	7850	tags=46%, list=22%, signal=36%
BP	GO:0046634	regulation of alpha-beta T cell activation	92	0.6596872	1.358802	0.001001	0.049713	0.049137	7890	tags=46%, list=22%, signal=36%
BP	GO:0032946	positive regulation of mononuclear cell proliferation	130	0.6596804	1.364904	0.000999	0.049713	0.049137	8427	tags=53%, list=23%, signal=41%
BP	GO:0050671	positive regulation of lymphocyte proliferation	129	0.6589942	1.363284	0.000999	0.049713	0.049137	8427	tags=53%, list=23%, signal=41%
BP	GO:0070665	positive regulation of leukocyte proliferation	138	0.6561437	1.358673	0.000999	0.049713	0.049137	8427	tags=53%, list=23%, signal=41%
BP	GO:0060337	type I interferon signaling pathway	94	0.6560576	1.351854	0.001001	0.049713	0.049137	7676	tags=49%, list=21%, signal=39%
BP	GO:0071357	cellular response to type I interferon	94	0.6560576	1.351854	0.001001	0.049713	0.049137	7676	tags=49%, list=21%, signal=39%
BP	GO:0071346	cellular response to interferon-gamma	175	0.6546146	1.356475	0.000999	0.049713	0.049137	8709	tags=52%, list=24%, signal=40%
BP	GO:0042098	T cell proliferation	182	0.653975	1.35579	0.000999	0.049713	0.049137	8186	tags=49%, list=22%, signal=39%
BP	GO:0002822	regulation of adaptive immune response based on somatic recombination of immune receptors built from immunoglobulin superfamily domains	144	0.6528501	1.35165	0.000999	0.049713	0.049137	7890	tags=46%, list=22%, signal=36%
BP	GO:0034341	response to interferon-gamma	193	0.6523002	1.353104	0.000999	0.049713	0.049137	8709	tags=53%, list=24%, signal=41%
BP	GO:0050727	regulation of inflammatory response	467	0.6521785	1.365954	0.000999	0.049713	0.049137	7886	tags=42%, list=22%, signal=34%
BP	GO:0034340	response to type I interferon	97	0.6519541	1.346777	0.000999	0.049713	0.049137	7676	tags=47%, list=21%, signal=38%

BP	GO:0035710	CD4-positive, alpha-beta T cell activation	91	0.6515976	1.342332	0.001001	0.049713	0.049137	8172	tags=48%, list=22%, signal=38%
BP	GO:0002819	regulation of adaptive immune response	159	0.6515057	1.349069	0.000999	0.049713	0.049137	7890	tags=46%, list=22%, signal=36%
BP	GO:0032944	regulation of mononuclear cell proliferation	207	0.6512452	1.353205	0.000999	0.049713	0.049137	8427	tags=52%, list=23%, signal=40%
BP	GO:0050670	regulation of lymphocyte proliferation	206	0.6507823	1.351968	0.000999	0.049713	0.049137	8427	tags=52%, list=23%, signal=40%
BP	GO:0016485	protein processing	325	0.6494435	1.35844	0.000999	0.049713	0.049137	7069	tags=33%, list=19%, signal=27%
BP	GO:0050870	positive regulation of T cell activation	201	0.6493045	1.347473	0.000999	0.049713	0.049137	8042	tags=47%, list=22%, signal=37%
BP	GO:0006898	receptor-mediated endocytosis	312	0.6491315	1.356691	0.000999	0.049713	0.049137	7641	tags=34%, list=21%, signal=27%
BP	GO:0070663	regulation of leukocyte proliferation	220	0.6485123	1.347769	0.000999	0.049713	0.049137	8427	tags=52%, list=23%, signal=40%
MF	GO:1903231	mRNA binding involved in posttranscriptional gene silencing	194	0.6464719	1.340905	0.000999	0.049713	0.049137	4380	tags=33%, list=12%, signal=29%
CC	GO:0016442	RISC complex	420	0.6444969	1.348642	0.000999	0.049713	0.049137	4186	tags=30%, list=11%, signal=26%
CC	GO:0031332	RNAi effector complex	420	0.6444969	1.348642	0.000999	0.049713	0.049137	4186	tags=30%, list=11%, signal=26%
BP	GO:1903039	positive regulation of leukocyte cell-cell adhesion	215	0.6443531	1.339529	0.000999	0.049713	0.049137	8446	tags=48%, list=23%, signal=37%
BP	GO:0002285	lymphocyte activation involved in immune response	180	0.6441855	1.335413	0.000999	0.049713	0.049137	8162	tags=46%, list=22%, signal=36%
MF	GO:0004896	cytokine receptor activity	96	0.6440235	1.330526	0.000999	0.049713	0.049137	7886	tags=53%, list=22%, signal=42%
BP	GO:0030217	T cell differentiation	239	0.6439342	1.34072	0.000999	0.049713	0.049137	8172	tags=44%, list=22%, signal=35%
BP	GO:0002703	regulation of leukocyte mediated immunity	201	0.6438224	1.336096	0.000999	0.049713	0.049137	8073	tags=48%, list=22%, signal=37%
MF	GO:0005125	cytokine activity	217	0.6421416	1.334551	0.000999	0.049713	0.049137	7732	tags=52%, list=21%, signal=41%
BP	GO:0045580	regulation of T cell differentiation	138	0.641424	1.328193	0.000999	0.049713	0.049137	7890	tags=44%, list=22%, signal=35%
BP	GO:0032943	mononuclear cell proliferation	272	0.6407049	1.3365	0.000999	0.049713	0.049137	8438	tags=50%, list=23%, signal=38%
BP	GO:0046651	lymphocyte proliferation	270	0.6400121	1.335934	0.000999	0.049713	0.049137	8438	tags=49%, list=23%, signal=38%

BP	GO:0097529	myeloid leukocyte migration	201	0.6398259	1.327803	0.000999	0.049713	0.049137	9232	tags=57%, list=25%, signal=43%
BP	GO:0035821	modification of morphology or physiology of other organism	162	0.639683	1.324468	0.000999	0.049713	0.049137	7222	tags=31%, list=20%, signal=25%
BP	GO:0002705	positive regulation of leukocyte mediated immunity	133	0.6390733	1.322429	0.000999	0.049713	0.049137	8073	tags=48%, list=22%, signal=38%
BP	GO:0070661	leukocyte proliferation	296	0.6359593	1.327764	0.000999	0.049713	0.049137	8446	tags=49%, list=23%, signal=38%
BP	GO:0030595	leukocyte chemotaxis	217	0.6349315	1.319566	0.000999	0.049713	0.049137	8284	tags=51%, list=23%, signal=39%
BP	GO:0050863	regulation of T cell activation	312	0.6347865	1.32671	0.000999	0.049713	0.049137	8186	tags=45%, list=22%, signal=35%
BP	GO:0007159	leukocyte cell-cell adhesion	332	0.6342109	1.326867	0.000999	0.049713	0.049137	8446	tags=46%, list=23%, signal=36%
BP	GO:0045619	regulation of lymphocyte differentiation	168	0.6338946	1.312511	0.000999	0.049713	0.049137	7890	tags=43%, list=22%, signal=34%
BP	GO:1903037	regulation of leukocyte cell-cell adhesion	299	0.6325629	1.321193	0.000999	0.049713	0.049137	8446	tags=45%, list=23%, signal=35%
BP	GO:0002699	positive regulation of immune effector process	214	0.6313505	1.312357	0.000999	0.049713	0.049137	8073	tags=43%, list=22%, signal=34%
BP	GO:0071219	cellular response to molecule of bacterial origin	208	0.6309448	1.310586	0.000999	0.049713	0.049137	8782	tags=45%, list=24%, signal=34%
BP	GO:0071222	cellular response to lipopolysaccharide	201	0.6307782	1.309026	0.000999	0.049713	0.049137	8782	tags=45%, list=24%, signal=34%
BP	GO:0030098	lymphocyte differentiation	352	0.6289953	1.315785	0.000999	0.049713	0.049137	8172	tags=42%, list=22%, signal=33%
BP	GO:0042110	T cell activation	462	0.6276911	1.314174	0.000999	0.049713	0.049137	8186	tags=44%, list=22%, signal=34%
MF	GO:0004984	olfactory receptor activity	419	0.626883	1.311661	0.000999	0.049713	0.049137	6447	tags=47%, list=18%, signal=39%
BP	GO:0050911	detection of chemical stimulus involved in sensory perception of smell	419	0.626883	1.311661	0.000999	0.049713	0.049137	6447	tags=47%, list=18%, signal=39%
BP	GO:0051604	protein maturation	394	0.6251619	1.308699	0.000999	0.049713	0.049137	6194	tags=24%, list=17%, signal=20%
BP	GO:0019882	antigen processing and presentation	223	0.6196317	1.288139	0.000999	0.049713	0.049137	8298	tags=35%, list=23%, signal=27%
BP	GO:0071216	cellular response to biotic stimulus	232	0.618862	1.288171	0.000999	0.049713	0.049137	8782	tags=41%, list=24%, signal=32%
BP	GO:0007608	sensory perception of smell	446	0.617198	1.291463	0.000999	0.049713	0.049137	6714	tags=46%, list=18%, signal=38%

BP	GO:0060326	cell chemotaxis	294	0.6151837	1.284256	0.000999	0.049713	0.049137	9232	tags=52%, list=25%, signal=39%
CC	GO:0043235	receptor complex	497	0.6137093	1.285896	0.000999	0.049713	0.049137	7504	tags=36%, list=21%, signal=29%
BP	GO:0050852	T cell receptor signaling pathway	199	0.6133372	1.272817	0.000999	0.049713	0.049137	8605	tags=33%, list=24%, signal=25%
BP	GO:0050907	detection of chemical stimulus involved in sensory perception	468	0.6132875	1.284324	0.000999	0.049713	0.049137	6447	tags=45%, list=18%, signal=38%
BP	GO:0022409	positive regulation of cell-cell adhesion	252	0.6123215	1.276422	0.000999	0.049713	0.049137	8661	tags=45%, list=24%, signal=35%
BP	GO:1902105	regulation of leukocyte differentiation	270	0.5963278	1.244749	0.000999	0.049713	0.049137	7966	tags=37%, list=22%, signal=29%
MF	GO:0005126	cytokine receptor binding	283	0.5929953	1.237203	0.000999	0.049713	0.049137	7602	tags=39%, list=21%, signal=31%
BP	GO:0032496	response to lipopolysaccharide	325	0.5897048	1.233485	0.000999	0.049713	0.049137	8782	tags=41%, list=24%, signal=32%
BP	GO:0022407	regulation of cell-cell adhesion	398	0.5836034	1.222019	0.000999	0.049713	0.049137	8461	tags=39%, list=23%, signal=31%
BP	GO:0042119	neutrophil activation	496	0.574194	1.203138	0.000999	0.049713	0.049137	10062	tags=43%, list=28%, signal=32%
